# Predictors of Symptom Scores in Myeloproliferative Neoplasms: A Real‐World Retrospective Cohort Study

**DOI:** 10.1002/cam4.71333

**Published:** 2025-11-29

**Authors:** Muhammad Ali Khan, Syed Arsalan Ahmed Naqvi, John K. Camoriano, Cecilia Y. Arana Yi, Jennifer T. Andres, Joshua L. Blocher, Heidi E. Kosiorek, Irbaz Bin Riaz, Jeanne M. Palmer

**Affiliations:** ^1^ Division of Hematology/Oncology, Department of Internal Medicine Mayo Clinic Phoenix Arizona USA; ^2^ Department of Quantitative Health Sciences Mayo Clinic Phoenix Arizona USA

## Abstract

**Background:**

Symptom management in myeloproliferative neoplasms (MPN) remains challenging despite advancements in disease‐directed therapies. This study assessed the impact of demographic, clinical, laboratory and treatment‐related variables on total symptom scores (TSS) and individual symptom scores in patients with polycythemia vera (PV), essential thrombocythemia (ET), and myelofibrosis (MF).

**Methods:**

A cohort of 553 patients (PV: 200; ET: 190; MF: 163) was identified through a retrospective chart review. Symptom scores, demographic information and clinical variables were captured at the time of the first TSS assessment. Laboratory results within 1 month and treatment information within 90 days prior to the symptom assessment were captured. Univariable linear regression, followed by multivariable regression analyses using a robust variance estimator were performed, with a *p* value < 0.05 indicating a significant association.

**Results:**

MF patients experienced the highest symptom burden and fatigue was the most reported symptom across all MPN subtypes. In PV, depression (*β*: 10.53; *p* = 0.001) was associated with a higher TSS whereas older age (−0.17; 0.01) and higher hemoglobin (−1.24; 0.003) were associated with a lower TSS. In ET, depression (*β*: 14.19; *p* < 0.001) was associated with a higher TSS, whereas in MF, depression (12.28; < 0.001) and higher WBC count (0.22; 0.02) were associated with a higher TSS. Depression and non‐White race in PV, depression in ET, and depression, low hemoglobin, and higher WBC count in MF were associated with multiple symptoms.

**Conclusions:**

Integrating depression screening and management and optimizing hematologic parameters alongside disease‐directed therapy is crucial to improving patient outcomes.

## 1 Introduction

Myeloproliferative neoplasms (MPN) are clonal myeloid malignancies broadly classified as Philadelphia chromosome (Ph)‐positive or Ph‐negative. The three most common Ph‐negative subtypes are polycythemia vera (PV), essential thrombocythemia (ET), and myelofibrosis (MF) [1]. Myelofibrosis may arise de novo as primary MF, or evolve from pre‐existing PV or ET, as secondary MF. Clinical presentation and long‐term outcomes vary by subtype: patients with PV and ET have an increased risk of thrombotic and hemorrhagic events, whereas those with MF typically experience a more aggressive course, a higher risk of transformation to acute myeloid leukemia, and worse prognosis [2]. Notably, all MPN patients experience a significant burden of symptoms [3]. These debilitating symptoms are often constitutional, such as fatigue, fever, night sweats, and weight loss; vascular, such as headache, concentration difficulties, and itching; or related to extramedullary hematopoiesis and splenomegaly, such as early satiety, abdominal discomfort, and bone pain.

The heterogeneity of symptom types and severity underscored the need for standardized symptom quantification in MPNs. An internet‐based survey of 1179 patients demonstrated significantly higher symptom burden in these patients compared to age‐matched controls, with MF showing the highest burden, and fatigue being the most prevalent symptom across all subtypes [4]. To enable systematic assessment, multiple patient‐reported outcome tools have been developed and validated. The original 20‐item Myelofibrosis Symptom Assessment Form (MF‐SAF) was designed to evaluate MF‐specific symptoms [5]. However, this tool did not capture the microvascular symptoms common in PV and ET. This led to the creation of the expanded 27‐item Myeloproliferative Neoplasm Symptom Assessment Form (MPN‐SAF) [6], later abbreviated into the 10‐item version for easier administration. The abbreviated MPN‐SAF captures the most representative and clinically relevant MPN symptoms: fatigue, early satiety, abdominal discomfort, inactivity, concentration difficulties, fever, night sweats, itching, bone pain, and weight loss, each rated on a 0–10 scale, with higher scores indicating greater severity. The total symptom score (TSS) is calculated by averaging the items and multiplying by 10 to yield a 0–100 scale. This 10‐item MPN‐SAF is now widely used in clinical practice and recommended by the National Comprehensive Cancer Network (NCCN) for routine symptom assessment in MPNs [7].

Higher symptom burden in MPNs is consistently associated with adverse clinical outcomes, increased thromboembolic risk, and poorer quality of life (QoL). The International Working Group of Myelofibrosis Research and Treatment identified constitutional symptoms as a negative prognostic factor for survival in MF [8]. A multivariable analysis from the German Study Group MPN (GSG‐MPN) Bioregistry showed that patients with higher fatigue and weight loss scores had a 1.8‐fold increased risk of death [9]. Similarly, the Quebec CMLMPN Research Group reported that an MPNSAF TSS > 20 was significantly associated with reduced overall survival [10]. In terms of thrombotic risk, a retrospective cohort of Turkish MPN patients demonstrated that those with thromboembolic events had significantly higher MPN‐SAF scores, with the strongest associations for fatigue, abdominal discomfort, inactivity, night sweats, pruritus, weight loss, and early satiety [11]. Similarly, a retrospective study in Italian patients found that the presence of clinical signs and symptoms was linked to a twofold increase in thrombosis risk [12]. Elevated symptom burden has also been shown to adversely affect quality of life (QoL). In a large international cohort of 1416 patients, fatigue and inactivity were the symptoms most strongly correlated with reduced QoL measured by the EORTC QLQC30 [13]. In another analysis of 402 patients, pain, fatigue, insomnia, and appetite loss were significantly associated with impaired QoL [14].

A ≥ 50% reduction in TSS is a key endpoint for assessing treatment efficacy in MPNs. Janus Kinase Inhibitors (JAK‐i), the cornerstone for managing symptoms and splenomegaly in MF [15, 16, 17, 18, 19, 20, 21, 22], have also demonstrated symptom improvement in PV [23, 24] and ET [25]. Cytoreductive agents provide similar benefits in PV and ET [26]. Non‐pharmacological interventions such as yoga [27, 28] and meditation [29] have also shown symptom improvement. Despite these interventions, most patients continue to experience debilitating symptoms, suggesting the influence of additional, unaddressed factors [30]. To this end, we have retrospectively evaluated the impact of demographic, clinical, and laboratory variables on MPN‐SAF TSS and individual symptom scores in a real‐world cohort of PV, ET, and MF patients, aiming to identify subgroups at higher risk for greater symptom burden and pinpoint clinically actionable targets to guide personalized symptom management.

## 2 Materials and Methods

### 2.1 Data Acquisition

Patients with clinical or hematopathological diagnosis of PV, ET, or MF who had documented symptom scores were identified through retrospective chart review. The MF group included both primary MF and secondary MF arising from PV (post‐PV MF) or ET (post‐ET MF). Symptom data were obtained from the 10‐item MPN‐SAF, including TSS and individual symptom scores for fatigue, early satiety, abdominal discomfort, inactivity, concentration difficulties, fever, night sweats, itching, bone pain, and weight loss. The clinical note from the first visit at which the MPN‐SAF was completed was used for symptom data extraction.

Demographic and clinical variables were recorded at the time of symptom assessment. Laboratory data were captured within 1 month of the assessment whereas treatment data included disease‐directed therapies received within 90 days prior to the assessment. Demographic variables included age, gender, race, and ethnicity. Clinical variables included time since definitive MPN diagnosis (in months), season of visit (Summer: June–September; Winter: October–May), spleen size (centimeters below the left costal margin on physical examination), depression (from documentation in the clinical note or the use of antidepressants i.e., selective serotonin uptake inhibitors, serotonin norepinephrine reuptake inhibitors, tricyclic antidepressants, atypical antidepressants with indication of a depressive disorder), time since depression diagnosis (in months), antidepressant treatment at follow‐up, obesity (from documentation in the clinical note or body mass index ≥ 30 kg/m^2^), and anemia (hemoglobin < 13.5 g/dL in men and < 12.5 g/dL in women). Laboratory variables included full blood count and serum chemistry. Disease‐directed therapies included cytoreductive therapy (phlebotomy, hydroxyurea, interferon, anagrelide) and JAK‐i (ruxolitinib, fedratinib, pacritinib, momelotinib). Definitions for the demographic, clinical, and laboratory variables are provided in Table S1.

### 2.2 Univariable Regression Analysis

The association of demographic, clinical, and laboratory variables with the TSS and individual symptom scores was evaluated using univariable linear regression. The model assumed a linear relationship between each variable and the symptom scores expressed as:

$$Ŷ=\beta_{0}+\beta_{1}X$$

where *β*_1_ represents the strength and direction of association between the variable (*X*) and the TSS or individual symptom scores (*Ŷ*). A *p* value < 0.05 indicated a statistically significant association.

### 2.3 Multivariable Regression Analysis

The multivariable regression analyses included variables that were significantly associated with the TSS or individual symptom scores from the univariable analyses. Variables with ≥ 85% of missing data were excluded, and complete case analysis was performed for the remaining variables (Table S2). Multicollinearity was evaluated using variance inflation factors (VIF) (Tables S3 and S4), and the variables with VIF ≥ 5 were excluded. Homoscedasticity was assessed by visual inspection of residual plots (Figure S1). Multivariable regression analyses were performed using a robust variance estimator, assuming a linear relationship between the predictor variables and symptom scores:

$$Ŷ=\beta_{0}+\beta_{1}X_{1}+\cdots +\beta_{n}X_{n}$$

where *β*_1_,…, *β*_*n*_ represent the strength and direction of the association between each variable (*X*_1_,…, *X*_*n*_) and the TSS or individual symptom scores (*Ŷ*), adjusted for all other variables in the model. Bootstrapping with 1000 iterations was performed to compute *p* values with *p* < 0.05, indicating a statistically significant association.

### 2.4 Ethics Statement

This study was performed in accordance with the Declaration of Helsinki of 1964 and subsequent versions. The study was approved by the Mayo Clinic Institutional Review Board (protocol number: 21‐002040). A waiver of informed consent was granted as the study involved a retrospective review of electronic health records.

## 3 Results

A total of 553 patients were identified: 200 with PV, 190 with ET, and 163 with MF (Table 1a). Among MF patients, 85 had primary MF, 38 had post‐PV MF, and 40 had post‐ET MF. The median age was 65 years (range: 27–93) for PV, 65 (20–96) for ET, and 69 (27–96) for MF. Patients with PV had an equal proportion of males and females (*n* = 100, 50%), ET patients were predominantly female (139, 73%), and MF patients were predominantly male (89, 55%). Most patients were White (PV: *n* = 183, 91%; ET: 170, 89%; MF: 151, 93%) or non‐Hispanic/Latino (PV: 185, 92%; ET: 183, 96%; MF: 149, 91%). The median time since MPN diagnosis was 36 months (range: 0–474 months) for PV, 46 (0–480) for ET, and 28 (0–455) for MF. Depression was more common in ET (*n* = 49, 26%) than in PV (42, 21%) or MF (30, 18%). Among patients with depression, 31 (15%) with PV, 33 (17%) with ET, and 23 (14%) with MF reported depression onset after MPN diagnosis. Antidepressant use at follow‐up was recorded in 17 (9%) PV, 21 (11%) ET, and 15 (9%) MF patients. Median hemoglobin levels were 13.5 g/dL (range: 7.1–23.3 g/dL) in PV, 13.1 g/dL (8.5–16.2 g/dL) in ET, and 10.7 g/dL (4.6–16.9 g/dL) in MF (Table 1b). Median white blood cell (WBC) counts were 6.9 × 10^9^/L (range: 1.8 × 10^9^–34.2 × 10^9^/L) in PV, 6.7 × 10^9^/L (2.1 × 10^9^–18.5 × 10^9^/L) in ET, and 8.2 × 10^9^/L (1.3 × 10^9^–131.7 × 10^9^/L) in MF.

**TABLE 1:** Patient distribution by demographic, clinical, and laboratory features.

(a) Distribution by demographic and clinical features			
	Polycythemia vera (*n* = 200)	Essential thrombocythemia (*n* = 190)	Myelofibrosis (*n* = 163)
Demographic features			
Age at follow up, median (range)	65 (27–93)	65 (20–96)	69 (27–96)
Gender, *n* (%)			
Female	100 (50)	139 (73)	74 (45)
Male	100 (50)	51 (27)	89 (55)
Race, *n* (%)			
White	183 (91)	170 (89)	151 (93)
Non‐white	17 (9)	20 (11)	12 (7)
Ethnicity, *n* (%)			
Hispanic or latino	15 (8)	7 (4)	14 (9)
Non‐hispanic or latino	185 (92)	183 (96)	149 (91)
Clinical features			
Months since MPN diagnosis preceding follow up, median (range)	36 (0–474)	46 (0–480)	28 (0–455)
Depression status at follow up, *n* (%)			
Depression	42 (21)	49 (26)	30 (18)
No depression	158 (79)	141 (74)	133 (82)
Months since depression report preceding follow up, median (range)	25 (0–169)	25 (0–220)	7 (0–170)
Patients with depression in relation to MPN diagnosis, *n* (%)			
Report before MPN	11 (6)	16 (9)	7 (4)
Report after MPN	31 (15)	33 (17)	23 (14)
Patients on antidepressants at follow up, *n* (%)			
Receiving antidepressants	17 (9)	21 (11)	15 (9)
Not receiving antidepressants	25 (12)	28 (15)	15 (9)
Obesity status at follow up, *n* (%)			
Obese	25 (12)	11 (6)	2 (1)
Not obese	175 (88)	179 (94)	161 (99)
Follow up visits by season, *n* (%)			
Summer	80 (40)	51 (27)	39 (24)
Winter	120 (60)	139 (73)	124 (76)
Splenomegaly at follow‐ up, *n* (%)			
Splenomegaly	9 (5)	1 (1)	50 (31)
No splenomegaly	120 (60)	119 (62)	64 (39)
Spleen size not available	71 (35)	70 (37)	49 (30)
Spleen size at follow‐up, median (range)	6 (2–12)	6 (−)	7 (1–30)
Anemia at follow‐up, *n* (%)			
Anemia	45 (23)	40 (21)	127 (78)
No anemia	155 (77)	150 (79)	36 (22)
Patients on treatment within 3 months preceding follow up, *n* (%)			
Cytoreductive therapy	155 (77)	150 (79)	36 (22)
Phlebotomy	98 (49)	—	—
Hydroxyurea	79 (40)	81 (43)	—
Interferon	37 (19)	32 (17)	—
Anagrelide	—	6 (3)	—
JAK‐inhibitors			
Ruxolitinib	30 (15)	—	68 (42)
Fedratinib	—	—	3 (2)
Momelotinib	—	—	3 (2)
Pacritinib	—	—	3 (2)

(b) Distribution by laboratory features						
	Polycythemia vera (*n* = 200)		Essential thrombocythemia (*n* = 190)		Myelofibrosis (*n* = 163)	
	Patients, *n* (%)	Value, median (range)	Patients, *n* (%)	Value, median (range)	Patients, *n* (%)	Value, median (range)
Hemoglobin (g/dL)	200 (100)	13.5 (7.1–23.3)	190 (100)	13.1 (8.5–16.2)	163 (100)	10.7 (4.6–16.9)
Hematocrit (%)	200 (100)	43.7 (27.2–72.3)	110 (58)	40.8 (26.9–49.3)	72 (44)	35.0 (21.7–51.4)
Platelet count (×10^9^/L)	200 (100)	349 (51–1084)	190 (100)	445 (160–2085)	162 (99)	237 (2–1589)
WBC count (×10^9^/L)	200 (100)	6.9 (1.8–34.2)	190 (100)	6.7 (2.1–18.5)	163 (100)	8.2 (1.3–131.7)
Circulating blasts (%)	1 (0.5)	1 (−)	1 (0.5)	0 (−)	53 (33)	2 (0–20)
Neutrophil count (×10^9^/L)	197 (99)	4.3 (1.1–29.4)	189 (99.5)	4.0 (1.0–12.7)	145 (89)	5.6 (0.2–93.5)
Non‐neutrophil WBC count (×10^9^/L)	197 (99)	2.2 (0.7–7.9)	189 (99.5)	2.4 (0.8–8.6)	145 (89)	2.5 (0.7–38.2)
Ferritin (μg/dL)	78 (39)	18 (4–704)	66 (35)	102 (11–577)	47 (29)	148 (8–1481)
Iron (μg/dL)	62 (31)	44.5 (10–178)	58 (31)	88.5 (23–176)	29 (18)	80 (11–171)
TIBC (μg/dL)	62 (31)	364.5 (207–463)	58 (31)	304 (218–503)	29 (18)	282 (159–426)
LDH (IU/L)	127 (64)	212 (124–555)	124 (65)	190.5 (122–848)	119 (73)	481 (144–2375)

*Note:* (a) Patient distribution by demographic and clinical features and (b) patient counts based on the availability of laboratory results and corresponding median laboratory results for patients.

Abbreviations: TIBC, total iron binding capacity; WBC, white blood cell.

**FIGURE 1:**
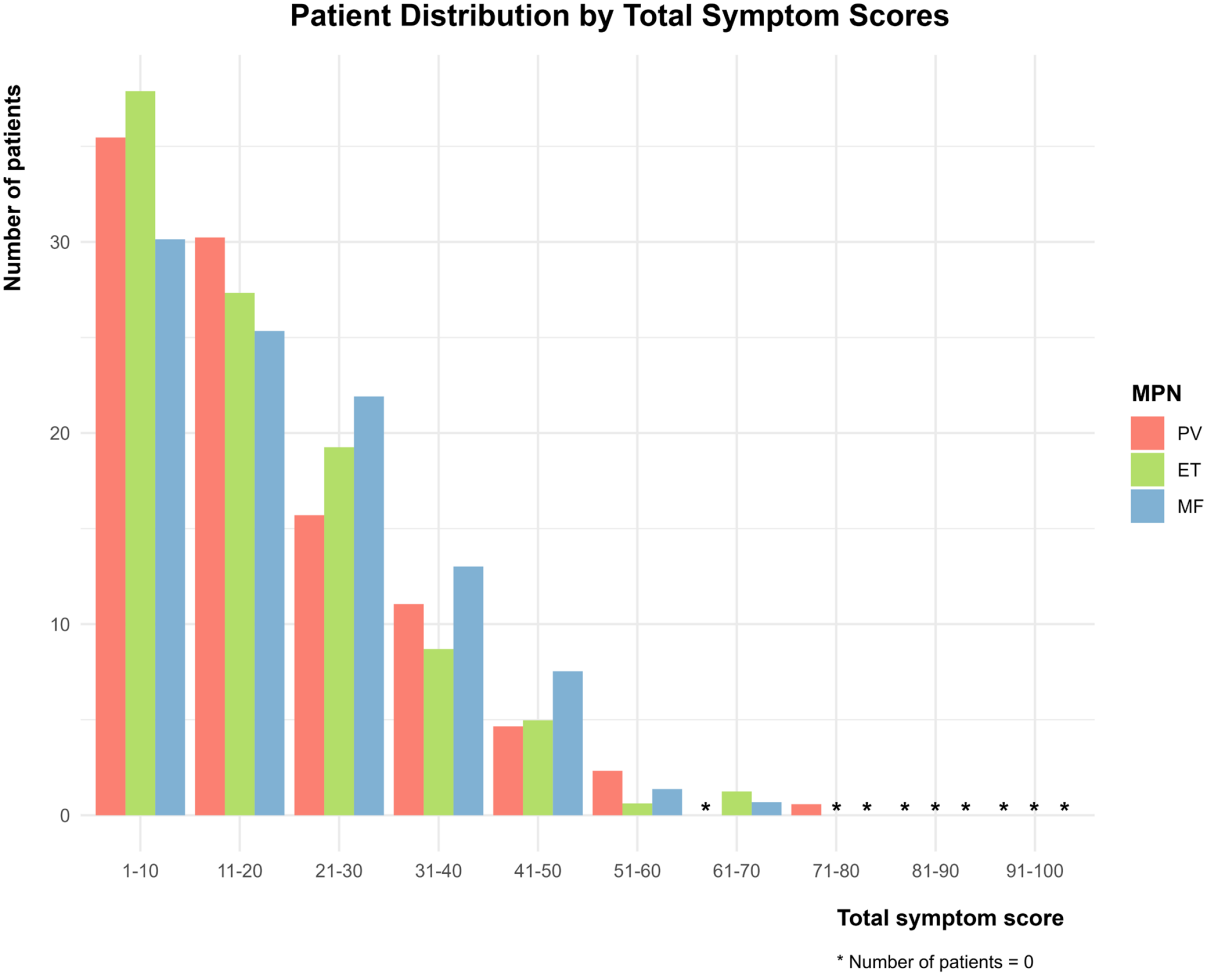
Frequency by total symptom scores. The distribution of patients by total symptom scores (TSS) ranging from 0 to 100. Twenty‐eight patients with PV, 29 with ET, and 17 with MF had no symptoms (TSS = 0). For each disease subtype, the number of patients decreases with an increasing symptom burden. ET, essential thrombocythemia; MF, myelofibrosis; MPN, myeloproliferative neoplasm; PV, polycythemia vera.

The median TSS was 12 (IQR: 4–23) in PV, 11 (4–23) in ET, and 14 (6–28) in MF (Table S5). Most patients had TSS between 1 and 40 (Figure 1) Fatigue was the most reported symptom across all MPN subtypes, whereas weight loss was the least reported symptom (Figure S2).

### 3.1 Univariable Regression Analysis

#### 3.1.1 Association With the Total Symptom Score

In PV, female gender (*β*: 5.95; *p* = 0.003) and depression (11.37; < 0.001) were associated with a higher TSS, whereas older age (−0.16; 0.04) and higher hemoglobin (−1.45; 0.006) were associated with a lower TSS (Table S6). In ET, female gender (*β*: 4.78; *p* = 0.04) and depression (15.47; < 0.001) were associated with a higher TSS. In MF, depression (*β*: 12.27; *p* < 0.001), splenomegaly (5.46; 0.04), higher WBC count (0.24; 0.001), higher neutrophil count (0.28; 0.01) and higher non‐neutrophil WBC count (0.59; 0.01) were associated with a higher TSS. Associations between demographic, clinical, and laboratory variables and TSS in patients with depression are shown in Table S9a.

#### 3.1.2 Association With the Individual Symptom Scores

In PV, depression was associated with fatigue (*β*: 1.56; *p* = 0.002), early satiety (1.61; < 0.001), abdominal discomfort (1.42; < 0.001), inactivity (1.71; 0.001) or concentration difficulties (2.36; < 0.001) (Table S7). Low hemoglobin was associated with fatigue (*β*: 0.25; *p* = 0.02), inactivity (0.24; 0.03) or night sweats (0.19; 0.04). Younger age was associated with abdominal discomfort (*β*: 0.03; *p* = 0.02), night sweats (0.04; 0.01), itching (0.03; 0.02) or bone pain (0.03; 0.01). Female gender was associated with abdominal discomfort (*β*: 0.89; *p* = 0.004), inactivity (1.00; 0.02), night sweats (0.73; 0.04), itching (0.91; 0.01) or bone pain (0.70; 0.01). Larger spleen size was associated with concentration difficulties (*β*: 0.82; *p* = 0.004) or itching (0.62; 0.01). A non‐White race was associated with fever (*β*: 1.4; *p* = 0.01) or weight loss (0.53; 0.01).

In ET, depression was associated with fatigue (*β*: 1.78; *p* < 0.001), early satiety (1.55; < 0.001), abdominal discomfort (1.74; < 0.001), inactivity (2.53; < 0.001), concentration difficulties (2.67; < 0.001), fever (1.41; < 0.001), night sweats (1.62; < 0.001), itching (1.55; < 0.001) or bone pain (0.57; 0.03).

In MF, depression was associated with fatigue (*β*: 1.98; *p* < 0.001), abdominal discomfort (0.96; 0.03), inactivity (2.05; 0.001), concentration difficulties (1.95; < 0.001), fever (0.96; 0.04), night sweats (1.61; 0.002) or bone pain (1.19; 0.01). Low hemoglobin was associated with fatigue (*β*: 0.43; *p* < 0.001), early satiety (0.20; 0.04) or inactivity (0.32; 0.01). Higher WBC count was associated with fatigue (*β*: 0.04; *p* = 0.02), early satiety (0.04; 0.002), bone pain (0.03; 0.004) or weight loss (0.43; < 0.001). Low iron was associated with fatigue (*β*: 0.04; *p* = 0.04) or weight loss (0.03; 0.03). Higher neutrophil count was associated with early satiety (*β*: 0.07; *p* = 0.001) or weight loss (0.06; < 0.001). Higher non‐neutrophil WBC was associated with early satiety (*β*: 0.13; *p* = 0.001), bone pain (0.09; 0.01) or weight loss (0.09; 0.001).

Associations between demographic, clinical, and laboratory variables and individual symptoms in patients with depression are shown in Table S9b.

### 3.2 Multivariable Regression Analysis

#### 3.2.1 Association With the Total Symptom Score

**FIGURE 2:**
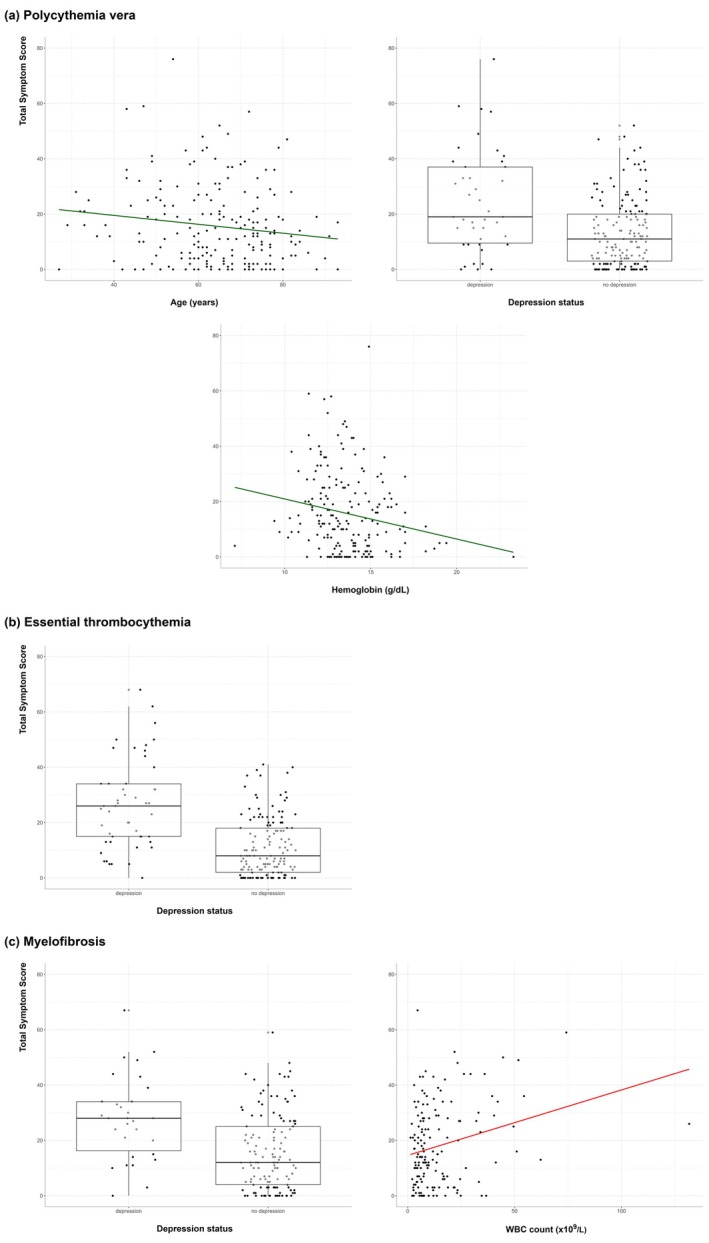
Predictors of total symptom scores from multivariable regression analysis. (a) Polycythemia vera. (b) Essential thrombocythemia. (c) Myelofibrosis. The predictors of the total symptom scores (TSS) from robust multivariable regression in patients with polycythemia vera (PV), essential thrombocythemia (ET), and myelofibrosis (MF). (a) In PV, patients with low hemoglobin had higher TSS than patients with higher hemoglobin, and patients with depression had higher TSS than patients without depression. (b) In ET, patients with depression had a higher TSS than patients without depression. (c) In MF, patients with depression had a higher TSS than patients without depression, and patients with a high white blood cell count had a higher TSS than patients with lower counts. WBC, white blood cell.

In PV, depression (*β*: 10.53; *p* = 0.001) was associated with a higher TSS, while old age (−0.17; 0.01) and higher hemoglobin (−1.24; 0.003) were associated with a lower TSS (Table 2, Figure 2). In ET, depression (*β*: 14.19; *p* < 0.001) was associated with higher TSS. In MF, depression (*β*: 12.28; *p* < 0.001) and higher WBC count (0.22; 0.02) were associated with a higher TSS. Associations between demographic, clinical, and laboratory variables with TSS in patients with depression are shown in Table S9c.

**TABLE 2:** Predictors of the total symptom scores from multivariable regression analysis.

Variable	Coefficient	*p*
Polycythemia vera		
Age (years)	**−0.17**	**0.01**
Gender: Female (vs. Male)	2.82	0.11
Depression at the time of the visit (vs. no depression)	**10.53**	**0.001**
Hemoglobin (g/dL)	**−1.24**	**0.003**
Essential thrombocythemia		
Gender: Female (vs. Male)	2.86	0.11
Depression at the time of the visit (vs. no depression)	**14.19**	**< 0.001**
Myelofibrosis		
Depression at the time of the visit (vs. no depression)	**12.28**	**< 0.001**
WBC count (×10^9^/L)	**0.22**	**0.02**

*Note:* The association of demographic, clinical, and laboratory variables with the total symptom score (TSS) from robust multivariable regression in patients with polycythemia vera (PV), essential thrombocythemia (ET), and myelofibrosis (MF). Depression in PV and ET, and depression and high white blood cell count in MF were significantly associated with high TSS. Old age and high hemoglobin in PV were significantly associated with low TSS. Bold values indicate a statistically significant association with high total symptom scores.

Abbreviation: WBC, white blood cell.

#### 3.2.2 Association With the Individual Symptom Scores

In PV, depression was associated with fatigue (*β*: 1.65; *p* = 0.001), inactivity (1.71; 0.03), and concentration difficulties (2.36; < 0.001). Non‐White race was associated with fever (*β*: 1.41; *p* = 0.008) and weight loss (0.53; 0.01) (Table S8).

In ET, depression was associated with fatigue (*β*: 1.98; *p* < 0.001), inactivity (2.78; < 0.001), concentration difficulties (2.67; < 0.001), night sweats (1.62; < 0.001), and bone pain (0.57; 0.03).

In MF, depression was associated with fatigue (*β*: 2.22; *p* < 0.001), inactivity (2.36; 0.001), concentration difficulties (1.95; < 0.001), fever (0.96; 0.04) and night sweats (1.61; 0.002). Low hemoglobin was associated with fatigue (*β*: 0.45; *p* = 0.001), early satiety (0.28; 0.004), inactivity (0.38; < 0.001). Higher WBC count was associated with fatigue (*β*: 0.03; *p* = 0.03) and early satiety (0.05; 0.004).

Association between demographic, clinical, and laboratory variables and individual symptoms in patients with depression is shown in Table S9d.

## 4 Discussion

Several demographic, clinical, and laboratory factors contributed to symptom burden in MPN patients. In PV, younger age and low hemoglobin were associated with higher TSS, while in MF, elevated white blood cell (WBC) count was associated with higher TSS. Depression was consistently associated with higher TSS across all MPN subtypes. Assessment of individual symptom scores highlighted the association of depression and non‐White race in PV, depression in ET, and depression, low hemoglobin, and elevated WBC count in MF with multiple symptoms. These findings propose systematic identification and management of anemia in PV, leukocytosis in MF, and depression across all MPN subtypes, as strategies to reduce symptom burden and improve patient outcomes.

The results build upon and extend prior observations. The MPN QOL International Working Group [31] reported a higher symptom burden among female patients, while the Quebec MPN Research Group [32] identified both younger and female patients as having a greater symptom burden, consistent with our findings. Notably, the overall symptom scores in our cohort (Table S2) were lower than those reported in the literature [6]. The relatively lower scores may reflect assessments performed in patients already receiving cytoreductive and JAK‐i therapy (Table 1), suggesting that such treatments may already have contributed to symptom reduction. Despite a lower overall symptom burden, the pattern of individual symptoms was comparable to previous reports [4, 6, 13, 14] with fatigue being the most prevalent and fever and weight loss, the least.

**FIGURE 3:**
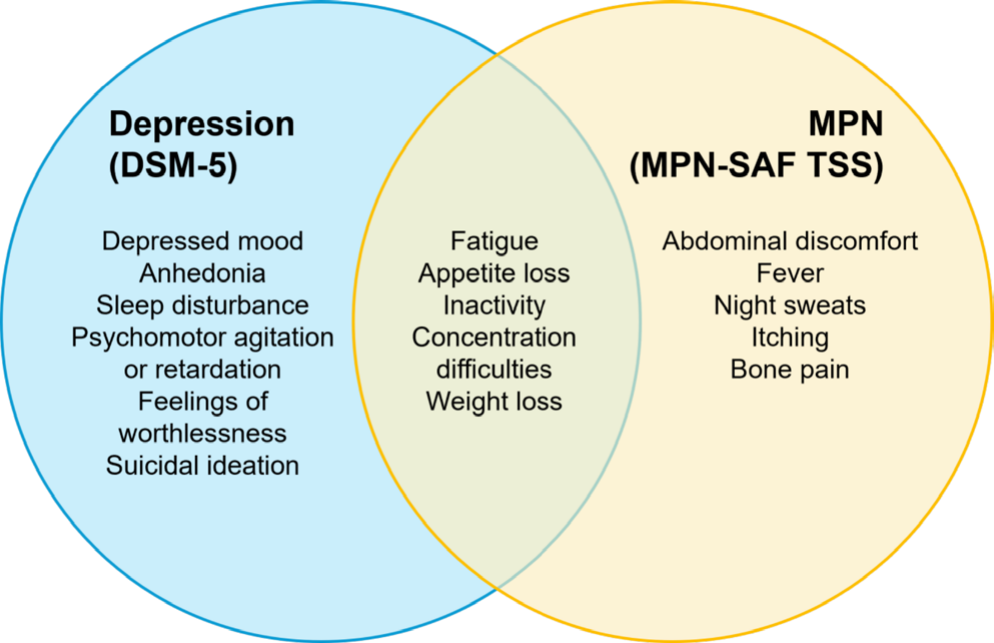
Overlapping symptoms between depression and MPNs. The symptoms unique to the DSM‐5 diagnostic criteria of depression and to the MPN‐SAF TSS assessment tool as well as the overlapping symptoms. This could possibly explain the association of depression with fatigue, inactivity and concentration difficulties in PV, ET and MF from the assessment of individual symptom scores. DSM‐5, diagnostic and statistical manual of mental disorders, fifth edition; MPN‐SAF TSS, myeloproliferative neoplasm‐symptom assessment form total symptom score.

Depression, in particular, was identified as a key contributor to symptom burden. This is partly due to the overlap between depressive symptoms, as defined in the Diagnostic and Statistical Manual of Mental Disorders, Fifth Edition (DSM‐5) [33], and the MPN‐SAF TSS items (Figure 3). Notably, fatigue, inactivity and concentration difficulties are core to both conditions and may result from shared biological mechanisms, such as chronic inflammation, cytokine dysregulation, hypothalamic–pituitary–adrenal (HPA) axis disturbances, and anemia, all of which are relevant in MPN pathophysiology. In depression, psychological factors, sleep disturbances, and reduced motivation can further amplify these symptoms, intensifying fatigue, limiting activity, and impairing cognition. Our findings confirmed the association of depression with fatigue, inactivity, and concentration difficulties across all MPN subtypes (Table S7). These overlapping manifestations can obscure the recognition of concurrent depression in patients with high disease‐related symptom burden, potentially delaying diagnosis and appropriate mental health interventions. This underscores the need for integrated screening for depression alongside MPN symptom assessment to enable early intervention.

Strengths of this study include the use of a real‐world patient cohort, a comprehensive analysis of both total and individual symptom scores, and employment of the validated MPN‐SAF tool for standardized symptom recording. The multivariable robust regression approach minimizes the influence of outliers and allows adjustment for key confounders, strengthening interpretability.

However, the findings of this study should be interpreted in the context of specific limitations. Limitations include underrepresentation of non‐White and Hispanic/Latino patients, restricting generalizability to these groups. The single time‐point assessment precluded evaluation of temporal symptom changes, requiring longitudinal studies to explore dynamic associations. The paradoxical link between lower hemoglobin and higher TSS in PV despite adjustment for iron deficiency requires validation in larger cohorts with comprehensive iron studies. Additionally, possible non‐linear associations between predictors and symptoms may be better captured using advanced machine learning techniques [34].

## 5 Conclusions

Depression, low hemoglobin, and elevated white blood cell counts emerged as key drivers of symptom burden in MPN patients, with fatigue, inactivity and concentration difficulties representing central overlapping features between depression and disease activity. These findings suggest integrating depression screening and management, and optimizing hematologic parameters, alongside disease‐directed therapy to meaningfully improve patient outcomes. Prospective multi‐institutional studies incorporating mental health interventions, and biologic profiling are warranted to validate these associations and guide personalized strategies for targeted symptom management.

## Author Contributions

Muhammad Ali Khan: conceptualization, methodology, data curation, formal analysis, visualization, writing – original draft, writing – review and editing, software, investigation. Syed Arsalan Ahmed Naqvi: methodology, formal analysis. John K. Camoriano: methodology, writing – review and editing. Cecilia Y. Arana Yi: methodology, writing – review and editing. Jennifer T. Andres: data curation. Joshua L. Blocher: data curation. Heidi E. Kosiorek: formal analysis, writing – review and editing. Irbaz Bin Riaz: conceptualization, methodology, supervision, writing – review and editing. Jeanne M. Palmer: conceptualization, methodology, supervision, writing – original draft, writing – review and editing, resources, investigation.

## Ethics Statement

This study was performed in accordance with the Declaration of Helsinki of 1964 and subsequent versions. The study was approved by the Mayo Clinic Institutional Review Board (protocol number: 21‐002040). A waiver of informed consent was granted as the study involved a retrospective review of electronic health records.

## Conflicts of Interest

The authors declare no conflicts of interest.

## Supporting information


**Data S1:** cam471333‐sup‐0001‐supinfo.docx.

## Data Availability

The data that support the findings of this study are available on request from the corresponding author. The data are not publicly available due to privacy or ethical restrictions.
