# Supplementary material for: Predictors of Symptom Scores in Myeloproliferative Neoplasms: A Real‐World Retrospective Cohort Study
Source: Cancer Med. 2025 Nov 29;14(23):e71333. doi: 10.1002/cam4.71333 (PMC12664104; doi:10.1002/cam4.71333)
Supplement: Supplementary file 1 — Data S1: cam471333‐sup‐0001‐supinfo.docx. [file CAM4-14-e71333-s001.docx]

**Supplementary Material**

- [Supplementary Table 1: Definitions of Demographic, Clinical, and Laboratory Variables](#_SDC,_Table_1:)
- [Supplementary Table 2: Patients Counts by Missing Variables](#_Supplementary_Table_2._1)
- [Supplementary Table 3: Multicollinearity within Predictors of Total Symptom Score](#_Supplementary_Table_3:_1)
- [Supplementary Table 4: Multicollinearity within Predictors of Individual Symptom Scores](#_Supplementary_Table_4:)
- [Supplementary Table 5: Distribution of Symptom Scores by MPN Subtype](#_Supplementary_Table_5:)
- [Supplementary Table 6: Predictors of the Total Symptom Scores from Univariable Regression Analyses](#_Supplementary_Table_2.)
- [Supplementary Table 7: Predictors of Individual Symptom Scores from Univariable Regression Analyses](#_Supplementary_Table_7:)
- [Supplementary Table 8: Predictors of Individual Symptom Scores from Multivariable Regression Analyses](#_Supplementary_Table_5._1)
- [Supplementary Table 9: Predictors of Symptom Scores in Patients with Depression](#_Supplementary_Table_9:)
- [Supplementary Figure 1: Residual Distribution for the Total Symptom Score](#_Supplementary_Figure_1:)
- [Supplementary Figure 2: Frequency of Individual Symptom Scores](#_Supplementary_Figure_2:)

# **Supplementary Table 1: Definitions of Demographic, Clinical, and Laboratory Variables**

| **Variable Category** | **Variable** | **Definition** |
| --- | --- | --- |
| *Demographic* | Age | Age (in years) at the time of the visit. |
|  | Sex | Biological sex assigned at birth. |
|  | Race | Race reported by the patients. |
|  | Ethnicity | Ethnicity reported by the patients. |
| *Clinical* | Months since diagnosis | Time in months between the definitive clinical or histopathologic MPN diagnosis and the index visit. |
|  | Depression | Clinical diagnosis of depression determined from the ICD codes or a mention of ongoing depressive disorder in the clinical note at the time of the visit. |
|  | Antidepressant consumption | Patients with depression who are receiving medications include selective serotonin reuptake inhibitors, serotonin-norepinephrine reuptake inhibitors, tricyclic antidepressants, and atypical antidepressants for indication of a depressive disorder. |
|  | Obesity | Clinical diagnosis of obesity determined from the ICD codes, calculated BMI ≥30 kg/m^2^ or a mention in the clinical note at the time of the visit. |
|  | Season at the time of the visit | Season at the time of the visit classified as Summers, which includes visits from June to September, and Winters, which includes visits from October to May. |
|  | Spleen size | Length (in centimeters) of the spleen below the LCM on physical examination. |
|  | Splenomegaly | Spleen size ≥5 cm below the LCM on physical examination |
|  | Anemia | Hemoglobin <13.5 g/dL in men and <12.5 g/dL in women. |
|  | Cytoreductive therapy | Phlebotomy, hydroxyurea and interferon in PV, and hydroxyurea, interferon and anagrelide in ET. |
|  | JAK-i therapy | Treatment with ruxolitinib, fedratinib, pacritinib or momelotinib. |
| *Laboratory* | Hemoglobin | Levels of hemoglobin in g/dL measured in the blood. |
|  | Hematocrit | Percentage of red blood cells by volume of the blood sample. |
|  | Platelet count | Number of platelets per liter of blood |
|  | WBC count | Number of white blood cells per liter of blood. |
|  | Neutrophil count | Number of neutrophils per liter of blood. |
|  | Non-neutrophil WBC count | Number of WBCs other than neutrophils per liter of blood. |
|  | Circulating blasts | Percentage of circulating blast cells in the blood. |
|  | Ferritin | Levels of ferritin in per deciliter of blood. |
|  | Iron | Levels of iron per deciliter of blood. |
|  | TIBC | Iron that can be bound to transferrin per deciliter of blood. |

Abbreviations: MPN: Myeloproliferative Neoplasms; ICD: International Classification of Diseases; LCM: left costal margin; PV: Polycythemia Vera; ET: Essential Thrombocythemia; MF: Myelofibrosis; JAK-i: Janus Kinase inhibitor; WBC: white blood cells; TIBC: total iron binding capacity

The table provides definitions of the demographic, clinical, and laboratory features of patients.

# **Supplementary Table 2: Patients Counts by Missing Variables**

| **Variable** | **Polycythemia Vera (n=200)** | **Essential Thrombocythemia (n=190)** | **Myelofibrosis (n=163)** |
| --- | --- | --- | --- |
| Demographic |  |  |  |
| Age, n (%) | 0 (0) | 0 (0) | 0 (0) |
| Gender, n (%) | 0 (0) | 0 (0) | 0 (0) |
| Race, n (%) | 0 (0) | 0 (0) | 0 (0) |
| Ethnicity, n (%) | 0 (0) | 0 (0) | 0 (0) |
| Clinical |  |  |  |
| Months since diagnosis, n (%) | 0 (0) | 0 (0) | 0 (0) |
| Depression status at the time of diagnosis, n (%) | 0 (0) | 0 (0) | 0 (0) |
| Antidepressant consumption, n (%)*** | 0 (0) | 0 (0) | 0 (0) |
| Obesity status at the time of diagnosis, n (%) | 0 (0) | 0 (0) | 0 (0) |
| Season at the time of the follow-up, n (%) | 0 (0) | 0 (0) | 0 (0) |
| Splenomegaly at the time of the follow-up, n (%) | 71 (35.5) | 70 (37) | 49 (30) |
| Spleen size at follow up, n (%) | 189 (94.5) | 189 (99) | 103 (63) |
| Anemia at the time of the follow-up, n (%) | 0 (0) | 0 (0) | 0 (0) |
| Cytoreductive therapy | 40 (20) | 75 (39) | 66 (40) |
| JAK-inhibitor therapy | 40 (20) | 75 (39) | 66 (40) |
| Laboratory |  |  |  |
| Hemoglobin (g/dL), n (%) | 0 (0) | 0 (0) | 0 (0) |
| Hematocrit (%), n (%) | 0 (0) | 80 (42) | 91 (56) |
| Platelet count (x10^9^/L), n (%) | 0 (0) | 0 (0) | 1 (1) |
| WBC count (x10^9^/L), n (%) | 0 (0) | 0 (0) | 0 (0) |
| Circulating blasts (%), n (%) | 199 (99.5) | 189 (99.5) | 110 (67.5) |
| Neutrophil count (x10^9^/L), n (%) | 3 (1.5) | 1 (0.5) | 18 (11) |
| Ferritin (µg/dL), n (%) | 122 (61) | 124 (65) | 116 (71) |
| Iron (µg/dL), n (%) | 138 (69) | 132 (69.5) | 134 (82) |
| TIBC (µg/dL), n (%) | 138 (69) | 132 (69.5) | 134 (82) |
| Non-neutrophil WBC count (x10^9^/L), n (%) | 3 (1.5) | 1 (0.5) | 18 (11) |
| Lactate dehydrogenase (IU/L), n (%) | 73 (37) | 73 (38) | 44 (27) |

Abbreviations: WBC: white blood cell; TIBC: total iron binding capacity.

The table shows the number of patients with missing assessments for each feature.

# **Supplementary Table 3: Multicollinearity within Predictors of Total Symptom Score**

| **Polycythemia vera** | |
| --- | --- |
| **Variable** | **VIF** |
| *Age (years)* | 1.00 |
| *Gender* | 1.12 |
| *Depression* | 1.01 |
| *Hemoglobin (g/dL)* | 1.12 |
| **Essential thrombocythemia** | |
| **Variable** | **VIF** |
| *Gender* | 1.02 |
| *Depression* | 1.02 |
| **Myelofibrosis** | |
| **Variable** | **VIF** |
| *Depression* | 1.07 |
| *Splenomegaly* | 1.03 |
| *WBC count (x10^9^/L)* | **25.24** |
| *Neutrophil count (x10^9^/L)* | **25.31** |

Abbreviations: WBC: white blood cell.

Bold values indicate features with high collinearity (VIF ≥5).

The table shows multicollinearity among features associated with the total symptom score on univariable regression. White blood cell and neutrophil counts showed high collinearity.

# **Supplementary Table 4: Multicollinearity within Predictors of Individual Symptom Scores**

**(a) Fatigue**

| **Polycythemia vera** | | | |
| --- | --- | --- | --- |
| **Variable** | | **VIF** | |
| *Depression* | | 1.00 | |
| *Anemia* | | 1.53 | |
| *Hemoglobin (g/dL)* | | 1.52 | |
| **Essential thrombocythemia** | | | |
| **Variable** | | **VIF** | |
| *Depression* | | 1.02 | |
| *Antidepressants* | | 1.02 | |
| *Anemia* | | 1.00 | |
| **Myelofibrosis** | | | |
| **Variable** | **VIF** | |  |
| *Age (years)* | | 1.11 | |
| *Depression* | | 1.43 | |
| *Anemia* | | 4.13 | |
| *Hemoglobin (g/dL)* | | 4.80 | |
| *WBC count (x10^9^/L)* | | 1.23 | |
| *Iron (µg/dL)* | | 1.69 | |

**(b) Early satiety**

| **Polycythemia vera** | |
| --- | --- |
| **Variable** | **VIF** |
| *Depression* | 1.00 |
| *WBC count (x10^9^/L)* | **23.87** |
| *Non-neutrophil WBC count (x10^9^/L)* | **23.87** |
| **Essential thrombocythemia** | |
| **Variable** | **VIF** |
| *Gender* | 1.09 |
| *Depression* | 1.03 |
| *Hemoglobin (g/dL)* | 1.08 |
| **Myelofibrosis** | |
| **Variable** | **VIF** |
| *Ethnicity* | 1.02 |
| *Hemoglobin (g/dL)* | 1.01 |
| *WBC count (x10^9^/L)* | **21.47** |
| *Neutrophil count (x10^9^/L)* | **21.44** |

**(c) Abdominal discomfort**

| **Polycythemia vera** | |
| --- | --- |
| **Variable** | **VIF** |
| *Age (years)* | 1.00 |
| *Gender* | 1.01 |
| *Depression* | 1.01 |
| **Essential thrombocythemia** | |
| **Variable** | **VIF** |
| *Depression* | 1.00 |
| *Obesity* | 1.00 |
| **Myelofibrosis** | |
| **Variable** | **VIF** |
| *Depression* | 1.00 |
| *Platelet count (x10^9^/L)* | 1.00 |

**(d) Inactivity**

| **Polycythemia vera** | |
| --- | --- |
| **Variable** | **VIF** |
| *Gender* | 1.12 |
| *Depression* | 1.01 |
| *Hemoglobin (g/dL)* | 1.11 |
| **Essential thrombocythemia** | |
| **Variable** | **VIF** |
| *Ethnicity* | 1.00 |
| *Time since diagnosis (months)* | 1.00 |
| *Depression* | 1.01 |
| **Myelofibrosis** | |
| **Variable** | **VIF** |
| *Depression* | 1.04 |
| *Splenomegaly* | 1.04 |
| *Hemoglobin (g/dL)* | 1.03 |

**(e) Fever**

| **Essential thrombocythemia** | |
| --- | --- |
| **Variable** | **VIF** |
| *Time since diagnosis (months)* | 1.06 |
| *Depression* | 1.01 |
| *Non-neutrophil WBC count (x10^9^/L)* | 1.05 |

**(f) Night sweats**

| **Polycythemia vera** | |
| --- | --- |
| **Variable** | **VIF** |
| *Age (years)* | 1.00 |
| *Gender* | 1.11 |
| *Hemoglobin (g/dL)* | 1.11 |
| **Myelofibrosis** | |
| **Variable** | **VIF** |
| *Depression* | 2.08 |
| *Spleen size (cm)* | 2.20 |
| *TIBC (µg/dL)* | 1.19 |

**(g) Itching**

| **Polycythemia vera** | |
| --- | --- |
| **Variable** | **VIF** |
| *Age (years)* | 1.00 |
| *Gender* | 1.00 |
| **Essential thrombocythemia** | |
| **Variable** | **VIF** |
| *Race* | 1.03 |
| *Depression* | 1.03 |
| **Myelofibrosis** | |
| **Variable** | **VIF** |
| *Gender* | 1.40 |
| *Race* | 1.09 |
| *Iron (µg/dL)* | 1.32 |

**(h) Bone pain**

| **Polycythemia vera** | | |
| --- | --- | --- |
| **Variable** | **VIF** | |
| *Age (years)* | 1.00 | |
| *Gender* | 1.00 | |
| **Myelofibrosis** | | |
| **Variable** | **VIF** |  |
| *Depression* | 1.09 | |
| *WBC count (x10^9^/L)* | 4.11 | |
| *Ferritin (µg/dL)* | 1.10 | |
| *Non-neutrophil WBC count (x10^9^/L)* | 4.16 | |

**(i) Weight loss**

| **Myelofibrosis** | |
| --- | --- |
| **Variable** | **VIF** |
| *WBC count (x10^9^/L)* | **14.25** |
| *Neutrophil count (x10^9^/L)* | **14.60** |
| *Iron (µg/dL)* | 1.10 |

Abbreviations: WBC: white blood cell.

Bold values indicate features with high collinearity (VIF ≥5).

The tables show multicollinearity among features associated with (a) fatigue, (b) early satiety, (c) abdominal discomfort, (d) inactivity, (e) fever, (f) night sweats, (g) itching, (h) bone pain and (i) weight loss, on univariable regression. White blood cell and neutrophil counts showed high collinearity.

# **Supplementary Table 5: Distribution of Symptom Scores by MPN Subtype**

| **Symptom Score** | **Polycythemia Vera** | | | **Essential Thrombocythemia** | | | **Myelofibrosis** | | |
| --- | --- | --- | --- | --- | --- | --- | --- | --- | --- |
|  | **Median** | **Range** | **IQR** | **Median** | **Range** | **IQR** | **Median** | **Range** | **IQR** |
| Total symptom score | 12 | 0-76 | 4-23 | 11 | 0-68 | 4-23 | 14 | 0-67 | 6-28 |
| Fatigue | 4 | 0-10 | 1-6 | 4 | 0-10 | 2-5 | 5 | 0-10 | 2-7 |
| Early satiety | 0 | 0-10 | 0-3 | 0 | 0-9 | 0-3.75 | 0 | 0-10 | 0-4 |
| Abdominal discomfort | 0 | 0-10 | 0-2 | 0 | 0-9 | 0-1 | 0 | 0-9 | 0-2 |
| Inactivity | 0 | 0-10 | 0-5 | 0 | 0-10 | 0-5 | 3 | 0-10 | 0-5 |
| Problems with concentration | 0 | 0-10 | 0-5 | 0 | 0-10 | 0-3 | 0 | 0-10 | 0-3 |
| Fever | 0 | 0-9 | 0-0 | 0 | 0-9 | 0-3 | 0 | 0-9 | 0-0 |
| Night sweats | 0 | 0-10 | 0-2.25 | 0 | 0-10 | 0-1.75 | 0 | 0-10 | 0-3 |
| Itching | 0 | 0-10 | 0-1.25 | 0 | 0-9 | 0-1 | 0 | 0-10 | 0-2 |
| Bone pain | 0 | 0-9 | 0-0 | 0 | 0-9 | 0-0 | 0 | 0-10 | 0-0 |
| Weight loss | 0 | 0-7 | 0-0 | 0 | 0-7 | 0-0 | 0 | 0-10 | 0-0 |

Abbreviations: MPN: myeloproliferative neoplasm IQR: interquartile range.

The table shows the median, range, and interquartile range of total and individual symptom scores in patients with polycythemia vera (PV), essential thrombocythemia (ET), and myelofibrosis (MF). The highest symptom burden is seen in the MF, followed by PV and ET. Among individual symptoms, fatigue showed the highest score across all MPN subtypes.

# **Supplementary Table 6: Predictors of the Total Symptom Scores from Univariable Regression Analyses**

| **Variable** | **Polycythemia vera** | | **Essential thrombocythemia** | | **Myelofibrosis** | |
| --- | --- | --- | --- | --- | --- | --- |
|  | **Coefficient** | ***p-value*** | **Coefficient** | ***p-value*** | **Coefficient** | ***p-value*** |
| **Demographic Features** |  |  |  |  |  |  |
| Age (years) | **-0.16** | ***0.04*** | -0.08 | *0.17* | 0.05 | *0.64* |
| Gender: Female (vs. Male) | **5.95** | ***0.004*** | **4.78** | ***0.04*** | 1.64 | *0.48* |
| Race: White (vs. non-White) | -3.10 | *0.40* | 4.57 | *0.17* | -1.85 | *0.68* |
| Ethnicity: Hispanic or Latino (vs. non-Hispanic and non-Latino) | -0.18 | *0.96* | -1.92 | *0.72* | 3.64 | *0.38* |
| **Clinical Features** |  |  |  |  |  |  |
| Time since diagnosis (months) | 0.002 | *0.87* | -0.02 | *0.13* | -0.03 | *0.13* |
| Depression at the time of the visit (vs. no depression) | **11.37** | ***<0.001*** | **15.47** | ***<0.001*** | **12.27** | ***<0.001*** |
| Antidepressants at the time of the visit (vs. no antidepressants) | 6.87 | *0.23* | 8.76 | *0.06* | -6.37 | *0.24* |
| Obesity at the time of the visit (vs. no obesity) | -1.55 | *0.62* | 5.90 | *0.18* | 4.60 | *0.66* |
| Season at the time of the visit: Summer (vs. Winter) | 0.80 | *0.71* | 0.55 | *0.81* | -1.53 | *0.57* |
| Splenomegaly at the time of the visit (vs. no splenomegaly) | 1.31 | *0.79* | -8.92 | *0.46* | **5.46** | ***0.04*** |
| Spleen size at the time of the visit (centimeters below the LCM) | 1.41 | *0.26* | - | *-* | 0.58 | *0.10* |
| Anemia (vs. no anemia) | 2.99 | *0.22* | 2.90 | *0.25* | 1.66 | *0.55* |
| Cytoreductive therapy (vs. no cytoreductive therapy) | -6.85 | *0.09* | -5.43 | *0.42* | - | - |
| JAK-inhibitor therapy (vs. no JAK-inhibitor therapy) | **11.90** | ***<0.001*** | - | - | 6.28 | *0.07* |
| **Laboratory Features** |  |  |  |  |  |  |
| Hemoglobin (g/dL) | **-1.45** | ***0.01*** | -0.49 | *0.48* | -0.88 | *0.09* |
| Hematocrit (%) | -0.06 | *0.72* | -0.12 | *0.66* | -0.12 | *0.64* |
| Platelet count (x10^9^/L) | 0.01 | *0.13* | 2.8x10^-6^ | *0.99* | -0.01 | *0.11* |
| WBC count (x10^9^/L) | 0.22 | *0.30* | 0.19 | *0.57* | **0.24** | ***0.001*** |
| Circulating blasts (%) | - | *-* | - | *-* | -0.15 | *0.83* |
| Neutrophil count (x10^9^/L) | 0.21 | *0.39* | 0.06 | *0.90* | **0.28** | ***0.01*** |
| Ferritin (µg/dL) | -3.5x10^-3^ | *0.84* | 0.02 | *0.23* | 2.8x10^-3^ | *0.72* |
| Iron (µg/dL) | -0.01 | *0.88* | 0.03 | *0.68* | -0.11 | *0.23* |
| TIBC (µg/dL) | -0.02 | *0.48* | -0.01 | *0.77* | 0.001 | *0.99* |
| Non-neutrophil WBC count (x10^9^/L) | 1.15 | *0.23* | 0.90 | *0.35* | **0.59** | ***0.01*** |
| Lactate dehydrogenase (IU/L) | 0.01 | *0.49* | 0.02 | *0.08* | 5.5x10^-3^ | *0.12* |

Abbreviations: WBC: white blood cell; TIBC: total iron binding capacity.

Bold values indicate a statistically significant association of a given feature with the total symptom scores.

The table shows the association of demographic, clinical, and laboratory features with the total symptom score (TSS) from univariable regression in patients with polycythemia vera (PV), essential thrombocythemia (ET), and myelofibrosis (MF). Female gender and depression in PV and ET, and depression, splenomegaly, high white blood cell, neutrophil, and non-neutrophil white blood cell counts were significantly associated with high TSS. Old age and high hemoglobin in PV were significantly associated with low TSS.

# **Supplementary Table 7: Predictors of Individual Symptom Scores from Univariable Regression Analyses**

**(a) Fatigue**

| **Variable** | **Polycythemia vera** | | **Essential thrombocythemia** | | **Myelofibrosis** | |
| --- | --- | --- | --- | --- | --- | --- |
|  | **Coefficient** | ***p-value*** | **Coefficient** | ***p-value*** | **Coefficient** | ***p-value*** |
| Demographic |  |  |  |  |  |  |
| *Age (years)* | -0.02 | *0.31* | -0.02 | *0.17* | **0.05** | ***0.02*** |
| *Gender: Female (vs. Male)* | 0.71 | *0.09* | 0.85 | *0.05* | -0.45 | *0.33* |
| *Race: White (vs. non-White)* | 0.97 | *0.20* | 0.82 | *0.20* | -0.32 | *0.72* |
| *Ethnicity: Hispanic or Latino (vs. non-Hispanic and non-Latino)* | 0.14 | *0.86* | 0.87 | *0.40* | -0.32 | *0.71* |
| Clinical |  |  |  |  |  |  |
| *Time since diagnosis (months)* | 1.2x10^-4^ | *0.97* | -1.4x10^-3^ | *0.62* | -3.7x10^-3^ | *0.32* |
| *Depression at the time of the visit (vs. no depression)* | **1.56** | ***0.002*** | **1.78** | ***<0.001*** | **1.98** | ***0.001*** |
| *Antidepressants at the time of the visit (vs. no antidepressants)* | 1.57 | *0.08* | **1.36** | ***0.04*** | -0.08 | *0.93* |
| *Obesity at the time of the visit (vs. no obesity)* | -0.37 | *0.56* | -0.01 | *0.99* | 2.24 | *0.29* |
| *Season at the time of the visit: Summer (vs. Winter)* | 0.37 | *0.39* | -0.28 | *0.52* | -0.01 | *0.99* |
| *Splenomegaly at the time of the visit (vs. no splenomegaly)* | -0.62 | *0.55* | -3.82 | *0.14* | 0.96 | *0.08* |
| *Spleen size at the time of the visit (centimeters below the LCM)* | 0.41 | *0.26* | - | *-* | 0.08 | *0.26* |
| *Anemia (vs. no anemia)* | **1.03** | ***0.04*** | **0.97** | ***0.04*** | **1.76** | ***0.002*** |
| *Cytoreductive therapy (vs. no cytoreductive therapy)* | -1.55 | *0.06* | -1.36 | *0.28* | -0.87 | *0.15* |
| *JAK-inhibitor therapy (vs. no JAK-inhibitor therapy)* | **1.93** | ***0.001*** | **1.24** | ***0.13*** | **1.82** | ***0.007*** |
| Laboratory |  |  |  |  |  |  |
| *Hemoglobin (g/dL)* | **-0.25** | ***0.02*** | -0.15 | *0.25* | **-0.43** | ***<0.001*** |
| *Hematocrit (%)* | -0.01 | *0.70* | -0.05 | *0.40* | -0.09 | *0.06* |
| *Platelet count (x10^9^/L)* | 5.6x10^-4^ | *0.62* | -4.9x10^-4^ | *0.53* | -1.5x10^-3^ | *0.11* |
| *WBC count (x10^9^/L)* | -0.02 | *0.61* | 0.02 | *0.71* | **0.04** | ***0.02*** |
| *Circulating blasts (%)* | - | *-* | - | *-* | -0.01 | *0.94* |
| *Neutrophil count (x10^9^/L)* | -0.04 | *0.43* | -0.01 | *0.88* | 0.04 | *0.06* |
| *Ferritin (µg/dL)* | 1.5x10^-3^ | *0.67* | 4.2x10^-3^ | *0.17* | 2.3x10^-3^ | *0.14* |
| *Iron (µg/dL)* | 0.01 | *0.24* | 0.02 | *0.11* | **-0.04** | ***0.04*** |
| *TIBC (µg/dL)* | -0.01 | *0.24* | 1.6x10^-3^ | *0.83* | -8.9x10^-3^ | *0.39* |
| *Non-neutrophil WBC count (x10^9^/L)* | 0.17 | *0.38* | 0.07 | *0.68* | 0.08 | *0.06* |
| *Lactate dehydrogenase (IU/L)* | -5.6x10^-7^ | *0.99* | 4.9x10^-3^ | *0.05* | **2.0x10^-3^** | ***0.004*** |

**(b) Early satiety**

| **Variable** | **Polycythemia vera** | | **Essential thrombocythemia** | | **Myelofibrosis** | |
| --- | --- | --- | --- | --- | --- | --- |
|  | **Coefficient** | ***p-value*** | **Coefficient** | ***p-value*** | **Coefficient** | ***p-value*** |
| Demographic |  |  |  |  |  |  |
| *Age (years)* | -0.02 | *0.26* | -0.01 | *0.20* | -0.01 | *0.53* |
| *Gender: Female (vs. Male)* | 0.58 | *0.12* | **1.07** | ***0.01*** | 0.31 | *0.48* |
| *Race: White (vs. non-White)* | -0.66 | *0.33* | -0.76 | *0.23* | -0.16 | *0.85* |
| *Ethnicity: Hispanic or Latino (vs. non-Hispanic and non-Latino)* | -0.09 | *0.90* | -0.40 | *0.70* | **1.86** | ***0.02*** |
| Clinical |  |  |  |  |  |  |
| *Time since diagnosis (months)* | 3.7x10^-3^ | *0.16* | -2.5x10^-3^ | *0.37* | -3.8x10^-3^ | *0.28* |
| *Depression at the time of the visit (vs. no depression)* | **1.61** | ***<0.001*** | **1.55** | ***0.001*** | 0.30 | *0.60* |
| *Antidepressants at the time of the visit (vs. no antidepressants)* | 0.66 | *0.52* | 0.41 | *0.66* | -1.55 | *0.15* |
| *Obesity at the time of the visit (vs. no obesity)* | 0.10 | *0.86* | 1.03 | *0.22* | 0.49 | *0.81* |
| *Season at the time of the visit: Summer (vs. Winter)* | 0.06 | *0.87* | 0.02 | *0.96* | 0.14 | *0.78* |
| *Splenomegaly at the time of the visit (vs. no splenomegaly)* | -0.42 | *0.66* | -1.46 | *0.56* | 1.00 | *0.06* |
| *Spleen size at the time of the visit (centimeters below the LCM)* | -0.23 | *0.63* | - | *-* | 0.04 | *0.58* |
| *Anemia (vs. no anemia)* | 0.38 | *0.40* | 0.74 | *0.13* | 0.17 | *0.76* |
| *Cytoreductive therapy (vs. no cytoreductive therapy)* | -1.01 | *0.17* | -0.83 | *0.51* | 0.67 | *0.29* |
| *JAK-inhibitor therapy (vs. no JAK-inhibitor therapy)* | **1.30** | ***0.01*** | 0.96 | *0.23* | -0.49 | *0.49* |
| Laboratory |  |  |  |  |  |  |
| *Hemoglobin (g/dL)* | -0.15 | *0.13* | **-0.27** | ***0.04*** | **-0.20** | ***0.04*** |
| *Hematocrit (%)* | -0.03 | *0.41* | -0.10 | *0.08* | -0.06 | *0.21* |
| *Platelet count (x10^9^/L)* | 1.2x10^-3^ | *0.24* | -1.9x10^-5^ | *0.98* | 2.6x10^-4^ | *0.77* |
| *WBC count (x10^9^/L)* | **0.09** | ***0.02*** | -0.05 | *0.45* | **0.04** | ***0.002*** |
| *Circulating blasts (%)* | - | *-* | - | *-* | -0.22 | *0.11* |
| *Neutrophil count (x10^9^/L)* | **0.11** | ***0.01*** | -0.11 | *0.20* | **0.07** | ***0.001*** |
| *Ferritin (µg/dL)* | -2.5x10^-3^ | *0.43* | -3.7x10^-4^ | *0.91* | -4.8x10^-4^ | *0.76* |
| *Iron (µg/dL)* | -2.3x10^-3^ | *0.82* | -2.6x10^-3^ | *0.82* | -0.01 | *0.51* |
| *TIBC (µg/dL)* | -3.2x10^-3^ | *0.61* | -0.01 | *0.18* | -3.2x10^-3^ | *0.72* |
| *Non-neutrophil WBC count (x10^9^/L)* | 0.12 | *0.49* | 0.18 | *0.32* | **0.13** | ***0.001*** |
| *Lactate dehydrogenase (IU/L)* | 5.2x10^-3^ | *0.14* | **6.3x10^-3^** | ***0.005*** | 8.0x10^-4^ | *0.27* |

**(c) Abdominal discomfort**

| **Variable** | **Polycythemia vera** | | **Essential thrombocythemia** | | **Myelofibrosis** | |
| --- | --- | --- | --- | --- | --- | --- |
|  | **Coefficient** | ***p-value*** | **Coefficient** | ***p-value*** | **Coefficient** | ***p-value*** |
| Demographic |  |  |  |  |  |  |
| *Age (years)* | **-0.03** | ***0.02*** | -0.01 | *0.23* | -0.01 | *0.37* |
| *Gender: Female (vs. Male)* | **0.89** | ***0.004*** | 0.73 | *0.06* | 0.59 | *0.09* |
| *Race: White (vs. non-White)* | -0.89 | *0.11* | 0.92 | *0.10* | 0.07 | *0.92* |
| *Ethnicity: Hispanic or Latino (vs. non-Hispanic and non-Latino)* | -0.70 | *0.24* | -1.27 | *0.17* | -0.26 | *0.67* |
| Clinical |  |  |  |  |  |  |
| *Time since diagnosis (months)* | 1.9x10^-3^ | *0.38* | -4.2x10^-3^ | *0.09* | 1.2x10^-3^ | *0.65* |
| *Depression at the time of the visit (vs. no depression)* | **1.42** | ***<0.001*** | **1.74** | ***<0.001*** | **0.96** | ***0.03*** |
| *Antidepressants at the time of the visit (vs. no antidepressants)* | 0.49 | *0.57* | 1.01 | *0.25* | 0.70 | *0.43* |
| *Obesity at the time of the visit (vs. no obesity)* | 0.25 | *0.61* | **2.08** | ***0.005*** | -1.33 | *0.40* |
| *Season at the time of the visit: Summer (vs. Winter)* | 0.13 | *0.69* | 0.21 | *0.60* | 0.30 | *0.47* |
| *Splenomegaly at the time of the visit (vs. no splenomegaly)* | 0.97 | *0.21* | -0.98 | *0.65* | 0.87 | *0.05* |
| *Spleen size at the time of the visit (centimeters below the LCM)* | -0.29 | *0.43* | - | *-* | 0.09 | *0.15* |
| *Anemia (vs. no anemia)* | 0.13 | *0.72* | 0.29 | *0.50* | -0.38 | *0.36* |
| *Cytoreductive therapy (vs. no cytoreductive therapy)* | -0.70 | *0.24* | -1.50 | *0.16* | -0.43 | *0.35* |
| *JAK-inhibitor therapy (vs. no JAK-inhibitor therapy)* | **1.03** | ***0.02*** | **1.46** | ***0.03*** | 0.73 | *0.15* |
| Laboratory |  |  |  |  |  |  |
| *Hemoglobin (g/dL)* | -0.14 | *0.09* | -0.10 | *0.39* | 0.05 | *0.56* |
| *Hematocrit (%)* | 0.02 | *0.52* | -0.04 | *0.51* | 0.02 | *0.53* |
| *Platelet count (x10^9^/L)* | 5.4x10^-4^ | *0.52* | -2.5x10^-4^ | *0.72* | **-1.5x10^-3^** | ***0.03*** |
| *WBC count (x10^9^/L)* | 0.03 | *0.37* | -0.03 | *0.56* | 3.8x10^-3^ | *0.73* |
| *Circulating blasts (%)* | - | *-* | - | *-* | -0.01 | *0.91* |
| *Neutrophil count (x10^9^/L)* | 0.03 | *0.37* | -0.04 | *0.58* | -4.7x10^-3^ | *0.77* |
| *Ferritin (µg/dL)* | -3.4x10^-3^ | *0.21* | 2.3x10^-4^ | *0.93* | -1.7x10^-3^ | *0.11* |
| *Iron (µg/dL)* | -8.8x10^-3^ | *0.30* | 2.8x10^-4^ | *0.98* | 0.01 | *0.47* |
| *TIBC (µg/dL)* | 2.0x10^-3^ | *0.72* | -0.01 | *0.26* | -5.2x10^-3^ | *0.45* |
| *Non-neutrophil WBC count (x10^9^/L)* | 0.08 | *0.60* | -0.01 | *0.94* | 2.9x10^-5^ | *0.99* |
| *Lactate dehydrogenase (IU/L)* | 1.5x10^-3^ | *0.59* | 2.6x10^-3^ | *0.24* | 3.7x10^-4^ | *0.50* |

**(d) Inactivity**

| **Variable** | **Polycythemia vera** | | **Essential thrombocythemia** | | **Myelofibrosis** | |
| --- | --- | --- | --- | --- | --- | --- |
|  | **Coefficient** | ***p-value*** | **Coefficient** | ***p-value*** | **Coefficient** | ***p-value*** |
| Demographic |  |  |  |  |  |  |
| *Age (years)* | 1.5x10^-3^ | *0.92* | -0.01 | *0.59* | 0.03 | *0.11* |
| *Gender: Female (vs. Male)* | **1.00** | ***0.02*** | 0.57 | *0.26* | -0.44 | *0.38* |
| *Race: White (vs. non-White)* | 0.68 | *0.38* | 0.98 | *0.18* | 1.00 | *0.30* |
| *Ethnicity: Hispanic or Latino (vs. non-Hispanic and non-Latino)* | 0.28 | *0.73* | **-2.67** | ***0.02*** | 1.62 | *0.07* |
| Clinical |  |  |  |  |  |  |
| *Time since diagnosis (months)* | -1.6x10^-3^ | *0.60* | **-6.5x10^-3^** | ***0.04*** | -2.6x10^-3^ | *0.52* |
| *Depression at the time of the visit (vs. no depression)* | **1.71** | ***0.001*** | **2.53** | ***<0.001*** | **2.05** | ***0.001*** |
| *Antidepressants at the time of the visit (vs. no antidepressants)* | 1.28 | *0.23* | 1.08 | *0.25* | -2.09 | *0.06* |
| *Obesity at the time of the visit (vs. no obesity)* | -0.31 | *0.63* | 0.74 | *0.44* | 1.43 | *0.53* |
| *Season at the time of the visit: Summer (vs. Winter)* | -0.26 | *0.55* | 0.10 | *0.84* | 0.05 | *0.94* |
| *Splenomegaly at the time of the visit (vs. no splenomegaly)* | -1.27 | *0.22* | -2.20 | *0.43* | **1.92** | ***0.001*** |
| *Spleen size at the time of the visit (centimeters below the LCM)* | - | *-* | - | *-* | 0.10 | *0.19* |
| *Anemia (vs. no anemia)* | 0.88 | *0.08* | 0.29 | *0.60* | 0.94 | *0.12* |
| *Cytoreductive therapy (vs. no cytoreductive therapy)* | **-1.88** | ***0.02*** | -0.56 | *0.70* | -1.15 | *0.09* |
| *JAK-inhibitor therapy (vs. no JAK-inhibitor therapy)* | **1.68** | ***0.006*** | **2.38** | ***0.01*** | **1.64** | ***0.03*** |
| Laboratory |  |  |  |  |  |  |
| *Hemoglobin (g/dL)* | **-0.24** | ***0.03*** | -0.06 | *0.69* | **-0.32** | ***0.01*** |
| *Hematocrit (%)* | -0.01 | *0.73* | -0.06 | *0.32* | -0.10 | *0.07* |
| *Platelet count (x10^9^/L)* | 2.0x10^-3^ | *0.08* | -1.1x10^-3^ | *0.25* | -1.2x10^-3^ | *0.23* |
| *WBC count (x10^9^/L)* | 0.07 | *0.12* | 0.05 | *0.47* | 0.03 | *0.05* |
| *Circulating blasts (%)* | - | *-* | - | *-* | -0.11 | *0.42* |
| *Neutrophil count (x10^9^/L)* | 0.07 | *0.19* | 0.06 | *0.56* | 0.05 | *0.06* |
| *Ferritin (µg/dL)* | 3.0x10^-3^ | *0.41* | 3.6x10^-3^ | *0.37* | 2.3x10^-3^ | *0.18* |
| *Iron (µg/dL)* | 2.6x10^-3^ | *0.82* | 0.01 | *0.46* | -0.03 | *0.09* |
| *TIBC (µg/dL)* | -4.6x10^-3^ | *0.52* | 0.01 | *0.53* | -8.1x10^-3^ | *0.45* |
| *Non-neutrophil WBC count (x10^9^/L)* | 0.35 | *0.08* | 0.10 | *0.64* | 0.08 | *0.12* |
| *Lactate dehydrogenase (IU/L)* | 2.0x10^-3^ | *0.58* | 1.7x10^-3^ | *0.56* | **1.6x10^-3^** | ***0.04*** |

**(e) Concentration difficulties**

| **Variable** | **Polycythemia vera** | | **Essential thrombocythemia** | | **Myelofibrosis** | |
| --- | --- | --- | --- | --- | --- | --- |
|  | **Coefficient** | ***p-value*** | **Coefficient** | ***p-value*** | **Coefficient** | ***p-value*** |
| Demographic |  |  |  |  |  |  |
| *Age (years)* | -0.02 | *0.11* | -0.01 | *0.51* | 0.01 | *0.46* |
| *Gender: Female (vs. Male)* | 0.45 | *0.27* | 0.47 | *0.28* | -0.14 | *0.72* |
| *Race: White (vs. non-White)* | -0.7 | *0.34* | 0.34 | *0.59* | 0.16 | *0.83* |
| *Ethnicity: Hispanic or Latino (vs. non-Hispanic and non-Latino)* | 0.02 | *0.98* | 0.89 | *0.38* | -0.31 | *0.65* |
| Clinical |  |  |  |  |  |  |
| *Time since diagnosis (months)* | -1.8x10^-3^ | *0.55* | 1.5x10^-3^ | *0.59* | -2.8x10^-3^ | *0.36* |
| *Depression at the time of the visit (vs. no depression)* | **2.36** | ***<0.001*** | **2.67** | ***<0.001*** | **1.95** | ***<0.001*** |
| *Antidepressants at the time of the visit (vs. no antidepressants)* | 1.51 | *0.14* | -0.33 | *0.72* | -0.30 | *0.78* |
| *Obesity at the time of the visit (vs. no obesity)* | 0.25 | *0.69* | 0.25 | *0.76* | 0.87 | *0.62* |
| *Season at the time of the visit: Summer (vs. Winter)* | -0.06 | *0.89* | 0.04 | *0.93* | -0.17 | *0.70* |
| *Splenomegaly at the time of the visit (vs. no splenomegaly)* | -0.23 | *0.82* | 3.10 | *0.24* | 0.59 | *0.19* |
| *Spleen size at the time of the visit (centimeters below the LCM)* | **0.82** | ***0.004*** | - | *-* | 0.05 | *0.41* |
| *Anemia (vs. no anemia)* | 0.45 | *0.36* | 0.35 | *0.47* | 0.08 | *0.87* |
| *Cytoreductive therapy (vs. no cytoreductive therapy)* | -1.27 | *0.10* | **-2.83** | ***0.02*** | **-1.09** | ***0.04*** |
| *JAK-inhibitor therapy (vs. no JAK-inhibitor therapy)* | **1.62** | ***0.005*** | **2.55** | ***7.5x10^-4^*** | **1.26** | ***0.04*** |
| Laboratory |  |  |  |  |  |  |
| *Hemoglobin (g/dL)* | -0.16 | *0.12* | 0.04 | *0.76* | -0.03 | *0.74* |
| *Hematocrit (%)* | 0.02 | *0.63* | 0.08 | *0.16* | 0.02 | *0.57* |
| *Platelet count (x10^9^/L)* | 1.1x10^-3^ | *0.31* | 1.2x10^-3^ | *0.13* | -8.7x10^-4^ | *0.26* |
| *WBC count (x10^9^/L)* | 4.4x10^-3^ | *0.92* | 0.08 | *0.22* | 0.02 | *0.09* |
| *Circulating blasts (%)* | - | *-* | - | *-* | 0.05 | *0.69* |
| *Neutrophil count (x10^9^/L)* | -0.01 | *0.83* | 0.14 | *0.10* | 0.01 | *0.41* |
| *Ferritin (µg/dL)* | 2.6x10^-3^ | *0.44* | 8.4x10^-4^ | *0.82* | 1.9x10^-4^ | *0.89* |
| *Iron (µg/dL)* | -1.3x10^-3^ | *0.90* | 0.01 | *0.65* | -0.03 | *0.09* |
| *TIBC (µg/dL)* | -2.8x10^-4^ | *0.97* | 4.2x10^-3^ | *0.64* | -1.6x10^-4^ | *0.99* |
| *Non-neutrophil WBC count (x10^9^/L)* | 0.23 | *0.22* | 0.11 | *0.55* | 0.06 | *0.10* |
| *Lactate dehydrogenase (IU/L)* | 1.6x10^-3^ | *0.65* | 9.7x10^-4^ | *0.69* | 4.9x10^-4^ | *0.41* |

**(f) Fever**

| **Variable** | **Polycythemia vera** | | **Essential thrombocythemia** | | **Myelofibrosis** | |
| --- | --- | --- | --- | --- | --- | --- |
|  | **Coefficient** | ***p-value*** | **Coefficient** | ***p-value*** | **Coefficient** | ***p-value*** |
| Demographic |  |  |  |  |  |  |
| *Age (years)* | 0.01 | *0.50* | -0.01 | *0.42* | -0.03 | *0.11* |
| *Gender: Female (vs. Male)* | 0.14 | *0.64* | 0.28 | *0.40* | 0.45 | *0.21* |
| *Race: White (vs. non-White)* | **-1.4** | ***0.01*** | 0.51 | *0.29* | 0.36 | *0.60* |
| *Ethnicity: Hispanic or Latino (vs. non-Hispanic and non-Latino)* | 0.34 | *0.55* | -0.14 | *0.85* | 0.64 | *0.32* |
| Clinical |  |  |  |  |  |  |
| *Time since diagnosis (months)* | 4.1x10^-3^ | *0.05* | 4.3x10^-3^ | ***0.04*** | -1.0x10^-3^ | *0.72* |
| *Depression at the time of the visit (vs. no depression)* | 0.71 | *0.05* | **1.41** | ***<0.001*** | **0.96** | ***0.04*** |
| *Antidepressants at the time of the visit (vs. no antidepressants)* | 1.33 | *0.08* | 0.82 | *0.28* | -0.35 | *0.72* |
| *Obesity at the time of the visit (vs. no obesity)* | 0.08 | *0.86* | 0.83 | *0.18* | -0.93 | *0.57* |
| *Season at the time of the visit: Summer (vs. Winter)* | 0.04 | *0.90* | 0.42 | *0.21* | -0.22 | *0.59* |
| *Splenomegaly at the time of the visit (vs. no splenomegaly)* | 0.92 | *0.27* | -0.71 | *0.68* | 0.57 | *0.20* |
| *Spleen size at the time of the visit (centimeters below the LCM)* | 0.08 | *0.82* | - | *-* | 5.9x10^-4^ | *0.99* |
| *Anemia (vs. no anemia)* | 0.20 | *0.58* | 0.25 | *0.49* | -3.3x10^-3^ | *0.99* |
| *Cytoreductive therapy (vs. no cytoreductive therapy)* | -0.85 | *0.14* | 0.38 | *0.70* | -0.42 | *0.45* |
| *JAK-inhibitor therapy (vs. no JAK-inhibitor therapy)* | **1.04** | ***0.01*** | 0.47 | *0.46* | -0.28 | *0.65* |
| Laboratory |  |  |  |  |  |  |
| *Hemoglobin (g/dL)* | -0.07 | *0.39* | -0.13 | *0.18* | 0.02 | *0.85* |
| *Hematocrit (%)* | 1.2x10^-3^ | *0.96* | -0.05 | *0.30* | 0.01 | *0.87* |
| *Platelet count (x10^9^/L)* | 2.6x10^-4^ | *0.80* | -1.4x10^-5^ | *0.98* | -2.5x10^-4^ | *0.73* |
| *WBC count (x10^9^/L)* | -5.9x10^-3^ | *0.85* | 0.09 | *0.06* | -6.5x10^-4^ | *0.95* |
| *Circulating blasts (%)* | - | *-* | - | *-* | 0.05 | *0.60* |
| *Neutrophil count (x10^9^/L)* | -0.02 | *0.54* | 0.02 | *0.76* | -0.01 | *0.36* |
| *Ferritin (µg/dL)* | 4.2x10^-4^ | *0.84* | 3.2x10^-3^ | *0.16* | -9.7x10^-4^ | *0.44* |
| *Iron (µg/dL)* | 3.0x10^-3^ | *0.59* | -0.01 | *0.16* | -0.01 | *0.43* |
| *TIBC (µg/dL)* | -4.5x10^-3^ | *0.19* | -0.01 | *0.35* | 4.0x10^-3^ | *0.58* |
| *Non-neutrophil WBC count (x10^9^/L)* | 0.15 | *0.26* | **0.30** | ***0.02*** | -0.02 | *0.57* |
| *Lactate dehydrogenase (IU/L)* | 3.9x10^-3^ | *0.18* | -1.6x10^-4^ | *0.93* | 7.4x10^-4^ | *0.18* |

**(g) Night sweats**

| **Variable** | **Polycythemia vera** | | **Essential thrombocythemia** | | **Myelofibrosis** | |
| --- | --- | --- | --- | --- | --- | --- |
|  | **Coefficient** | ***p-value*** | **Coefficient** | ***p-value*** | **Coefficient** | ***p-value*** |
| Demographic |  |  |  |  |  |  |
| *Age (years)* | **-0.04** | ***0.01*** | 3.0x10^-3^ | *0.78* | -1.2x10^-3^ | *0.95* |
| *Gender: Female (vs. Male)* | **0.73** | ***0.04*** | 0.52 | *0.19* | 0.40 | *0.34* |
| *Race: White (vs. non-White)* | 0.10 | *0.87* | 0.96 | *0.10* | -0.53 | *0.50* |
| *Ethnicity: Hispanic or Latino (vs. non-Hispanic and non-Latino)* | 0.77 | *0.26* | -0.02 | *0.98* | -0.51 | *0.49* |
| Clinical |  |  |  |  |  |  |
| *Time since diagnosis (months)* | -1.3x10^-3^ | *0.61* | -3.6x10^-3^ | *0.16* | -4.3x10^-3^ | *0.19* |
| *Depression at the time of the visit (vs. no depression)* | 0.78 | *0.08* | **1.62** | ***<0.001*** | **1.61** | ***0.002*** |
| *Antidepressants at the time of the visit (vs. no antidepressants)* | 1.25 | *0.20* | 1.83 | *0.05* | 0.50 | *0.60* |
| *Obesity at the time of the visit (vs. no obesity)* | -0.53 | *0.33* | 0.06 | *0.94* | 0.75 | *0.69* |
| *Season at the time of the visit: Summer (vs. Winter)* | 0.63 | *0.09* | -0.18 | *0.66* | -0.86 | *0.08* |
| *Splenomegaly at the time of the visit (vs. no splenomegaly)* | 0.14 | *0.88* | -1.26 | *0.60* | -0.13 | *0.80* |
| *Spleen size at the time of the visit (centimeters below the LCM)* | -0.08 | *0.77* | - | *-* | **0.18** | ***0.003*** |
| *Anemia (vs. no anemia)* | 0.43 | *0.32* | -0.13 | *0.76* | -0.39 | *0.44* |
| *Cytoreductive therapy (vs. no cytoreductive therapy)* | -0.46 | *0.52* | 1.54 | *0.19* | -2.7x10^-3^ | *0.99* |
| *JAK-inhibitor therapy (vs. no JAK-inhibitor therapy)* | **1.31** | ***0.01*** | 0.77 | *0.31* | 0.56 | *0.36* |
| Laboratory |  |  |  |  |  |  |
| *Hemoglobin (g/dL)* | **-0.19** | ***0.04*** | 0.05 | *0.66* | 0.03 | *0.71* |
| *Hematocrit (%)* | -0.02 | *0.42* | 0.05 | *0.32* | 0.04 | *0.35* |
| *Platelet count (x10^9^/L)* | 1.3x10^-3^ | *0.18* | -3.8x10^-5^ | *0.96* | -1.1x10^-3^ | *0.19* |
| *WBC count (x10^9^/L)* | 0.05 | *0.22* | 0.03 | *0.64* | 0.02 | *0.25* |
| *Circulating blasts (%)* | - | *-* | - | *-* | 0.03 | *0.78* |
| *Neutrophil count (x10^9^/L)* | 0.06 | *0.16* | 3.1x10^-4^ | *0.99* | 0.02 | *0.29* |
| *Ferritin (µg/dL)* | -1.9x10^-3^ | *0.46* | 3.6x10^-3^ | *0.27* | -1.1x10^-3^ | *0.38* |
| *Iron (µg/dL)* | -7.5x10^-3^ | *0.36* | -2.4x10^-3^ | *0.84* | -0.01 | *0.40* |
| *TIBC (µg/dL)* | -3.2x10^-3^ | *0.55* | -1.4x10^-3^ | *0.86* | **0.02** | ***0.03*** |
| *Non-neutrophil WBC count (x10^9^/L)* | -1.5x10^-3^ | *0.99* | 0.07 | *0.66* | 0.03 | *0.51* |
| *Lactate dehydrogenase (IU/L)* | 1.0x10^-3^ | *0.75* | 1.7x10^-3^ | *0.47* | 5.7x10^-4^ | *0.35* |

**(h) Itching**

| **Variable** | **Polycythemia vera** | | **Essential thrombocythemia** | | **Myelofibrosis** | |
| --- | --- | --- | --- | --- | --- | --- |
|  | **Coefficient** | ***p-value*** | **Coefficient** | ***p-value*** | **Coefficient** | ***p-value*** |
| Demographic |  |  |  |  |  |  |
| *Age (years)* | **-0.03** | ***0.02*** | -4.7x10^-3^ | *0.65* | -0.02 | *0.22* |
| *Gender: Female (vs. Male)* | **0.91** | ***0.01*** | 0.21 | *0.59* | **0.94** | ***0.01*** |
| *Race: White (vs. non-White)* | -0.5 | *0.45* | **1.19** | ***0.04*** | **-1.78** | ***0.01*** |
| *Ethnicity: Hispanic or Latino (vs. non-Hispanic and non-Latino)* | -0.66 | *0.32* | 1.41 | *0.13* | 0.72 | *0.26* |
| Clinical |  |  |  |  |  |  |
| *Time since diagnosis (months)* | -2.2x10^-3^ | *0.38* | -2.3x10^-3^ | *0.36* | -4.1x10^-3^ | *0.15* |
| *Depression at the time of the visit (vs. no depression)* | 0.68 | *0.11* | **1.55** | ***<0.001*** | 0.69 | *0.14* |
| *Antidepressants at the time of the visit (vs. no antidepressants)* | -0.70 | *0.45* | 1.67 | *0.06* | -0.29 | *0.79* |
| *Obesity at the time of the visit (vs. no obesity)* | -0.91 | *0.08* | 0.54 | *0.47* | -1.29 | *0.43* |
| *Season at the time of the visit: Summer (vs. Winter)* | -0.15 | *0.68* | -0.16 | *0.69* | -0.56 | *0.19* |
| *Splenomegaly at the time of the visit (vs. no splenomegaly)* | 0.57 | *0.51* | -1.29 | *0.60* | -0.71 | *0.08* |
| *Spleen size at the time of the visit (centimeters below the LCM)* | **0.62** | ***0.01*** | - | *-* | 0.05 | *0.31* |
| *Anemia (vs. no anemia)* | -0.61 | *0.14* | 0.36 | *0.40* | -0.58 | *0.18* |
| *Cytoreductive therapy (vs. no cytoreductive therapy)* | 0.94 | *0.15* | -0.79 | *0.28* | 0.58 | *0.25* |
| *JAK-inhibitor therapy (vs. no JAK-inhibitor therapy)* | 0.72 | *0.13* | 0.79 | *0.28* | -0.49 | *0.39* |
| Laboratory |  |  |  |  |  |  |
| *Hemoglobin (g/dL)* | -0.15 | *0.09* | 0.08 | *0.49* | 0.09 | *0.29* |
| *Hematocrit (%)* | -0.03 | *0.27* | 0.03 | *0.54* | 0.02 | *0.56* |
| *Platelet count (x10^9^/L)* | 4.3x10^-4^ | *0.65* | -2.7x10^-4^ | *0.70* | 1.1x10^-4^ | *0.88* |
| *WBC count (x10^9^/L)* | 0.01 | *0.79* | -0.05 | *0.41* | 0.01 | *0.31* |
| *Circulating blasts (%)* | - | *-* | - | *-* | -0.02 | *0.88* |
| *Neutrophil count (x10^9^/L)* | 0.01 | *0.86* | -0.06 | *0.43* | 0.01 | *0.58* |
| *Ferritin (µg/dL)* | -1.5x10^-3^ | *0.64* | 6.1x10^-3^ | *0.07* | -1.1x10^-3^ | *0.35* |
| *Iron (µg/dL)* | -7.0x10^-3^ | *0.49* | 8.9x10^-3^ | *0.47* | **0.03** | ***0.01*** |
| *TIBC (µg/dL)* | -7.3x10^-4^ | *0.91* | 3.2x10^-3^ | *0.70* | 4.4x10^-3^ | *0.95* |
| *Non-neutrophil WBC count (x10^9^/L)* | 0.09 | *0.59* | -0.05 | *0.76* | 0.03 | *0.31* |
| *Lactate dehydrogenase (IU/L)* | -1.3x10^-3^ | *0.65* | 2.8x10^-3^ | *0.18* | 3.6x10^-4^ | *0.54* |

**(i) Bone pain**

| **Variable** | **Polycythemia vera** | | **Essential thrombocythemia** | | **Myelofibrosis** | |
| --- | --- | --- | --- | --- | --- | --- |
|  | **Coefficient** | ***p-value*** | **Coefficient** | ***p-value*** | **Coefficient** | ***p-value*** |
| Demographic |  |  |  |  |  |  |
| *Age (years)* | **-0.03** | ***0.01*** | -0.01 | *0.06* | 0.01 | *0.33* |
| *Gender: Female (vs. Male)* | **0.70** | ***0.01*** | 0.30 | *0.27* | 0.44 | *0.20* |
| *Race: White (vs. non-White)* | 0.09 | *0.85* | -0.14 | *0.72* | 0.43 | *0.52* |
| *Ethnicity: Hispanic or Latino (vs. non-Hispanic and non-Latino)* | -0.23 | *0.65* | -0.49 | *0.44* | 0.27 | *0.66* |
| Clinical |  |  |  |  |  |  |
| *Time since diagnosis (months)* | -1.9x10^-3^ | *0.30* | -2.4x10^-3^ | *0.16* | -3.6x10^-3^ | *0.18* |
| *Depression at the time of the visit (vs. no depression)* | 0.55 | *0.09* | **0.57** | ***0.03*** | **1.19** | ***0.01*** |
| *Antidepressants at the time of the visit (vs. no antidepressants)* | 0.30 | *0.69* | -0.04 | *0.95* | -2.09 | *0.06* |
| *Obesity at the time of the visit (vs. no obesity)* | -0.24 | *0.55* | 0.46 | *0.36* | **3.14** | ***0.04*** |
| *Season at the time of the visit: Summer (vs. Winter)* | 0.34 | *0.21* | 0.24 | *0.38* | 0.10 | *0.80* |
| *Splenomegaly at the time of the visit (vs. no splenomegaly)* | 0.34 | *0.55* | -0.40 | *0.79* | 0.25 | *0.52* |
| *Spleen size at the time of the visit (centimeters below the LCM)* | 0.11 | *0.71* | - | *-* | -0.02 | *0.67* |
| *Anemia (vs. no anemia)* | -0.16 | *0.62* | 0.16 | *0.58* | -0.06 | *0.88* |
| *Cytoreductive therapy (vs. no cytoreductive therapy)* | 0.21 | *0.68* | 0.55 | *0.51* | -0.95 | *0.07* |
| *JAK-inhibitor therapy (vs. no JAK-inhibitor therapy)* | 0.71 | *0.05* | 0.89 | *0.09* | **1.31** | ***0.02*** |
| Laboratory |  |  |  |  |  |  |
| *Hemoglobin (g/dL)* | -0.11 | *0.11* | 0.01 | *0.94* | -0.05 | *0.57* |
| *Hematocrit (%)* | 0.01 | *0.65* | -0.01 | *0.73* | 0.01 | *0.73* |
| *Platelet count (x10^9^/L)* | 8.2x10^-4^ | *0.25* | 6.4x10^-4^ | *0.18* | -9.7x10^-4^ | *0.17* |
| *WBC count (x10^9^/L)* | 1.9x10^-4^ | *0.99* | 0.04 | *0.36* | **0.03** | ***<0.001*** |
| *Circulating blasts (%)* | - | *-* | - | *-* | -0.02 | *0.87* |
| *Neutrophil count (x10^9^/L)* | -3.8x10^-3^ | *0.90* | 0.04 | *0.40* | 0.03 | *0.06* |
| *Ferritin (µg/dL)* | -2.2x10^-3^ | *0.35* | -8.4x10^-4^ | *0.70* | **2.1x10^-3^** | ***0.04*** |
| *Iron (µg/dL)* | -2.1x10^-3^ | *0.79* | 3.8x10^-3^ | *0.64* | 6.2x10^-4^ | *0.96* |
| *TIBC (µg/dL)* | 1.2x10^-3^ | *0.80* | -1.1x10^-3^ | *0.83* | -4.4x10^-3^ | *0.49* |
| *Non-neutrophil WBC count (x10^9^/L)* | 0.07 | *0.58* | 0.12 | *0.28* | **0.09** | ***0.01*** |
| *Lactate dehydrogenase (IU/L)* | 2.6x10^-4^ | *0.91* | -8.1x10^-4^ | *0.57* | 4.2x10^-4^ | *0.47* |

**(j) Weight loss**

| **Variable** | **Polycythemia vera** | | **Essential thrombocythemia** | | **Myelofibrosis** | |
| --- | --- | --- | --- | --- | --- | --- |
|  | **Coefficient** | ***p-value*** | **Coefficient** | ***p-value*** | **Coefficient** | ***p-value*** |
| Demographic |  |  |  |  |  |  |
| *Age (years)* | 0.01 | *0.17* | 6.7x10^-4^ | *0.87* | 0.01 | *0.37* |
| *Gender: Female (vs. Male)* | -0.16 | *0.19* | -0.28 | *0.08* | -0.45 | *0.13* |
| *Race: White (vs. non-White)* | **-0.53** | ***0.01*** | -0.03 | *0.91* | -0.60 | *0.29* |
| *Ethnicity: Hispanic or Latino (vs. non-Hispanic and non-Latino)* | 0.55 | *0.02* | -0.23 | *0.53* | -0.29 | *0.58* |
| Clinical |  |  |  |  |  |  |
| *Time since diagnosis (months)* | 2.6x10^-4^ | *0.76* | -1.1x10^-3^ | *0.28* | -3.7x10^-4^ | *0.87* |
| *Depression at the time of the visit (vs. no depression)* | -0.02 | *0.89* | 0.11 | *0.51* | 0.58 | *0.13* |
| *Antidepressants at the time of the visit (vs. no antidepressants)* | -0.41 | *0.30* | 0.06 | *0.87* | -0.96 | *0.28* |
| *Obesity at the time of the visit (vs. no obesity)* | -0.05 | *0.81* | -0.24 | *0.43* | -0.70 | *0.60* |
| *Season at the time of the visit: Summer (vs. Winter)* | -0.06 | *0.64* | 0.04 | *0.81* | -0.41 | *0.24* |
| *Splenomegaly at the time of the visit (vs. no splenomegaly)* | -0.19 | *0.56* | -0.12 | *0.85* | 0.08 | *0.80* |
| *Spleen size at the time of the visit (centimeters below the LCM)* | -0.03 | *0.44* | - | *-* | 0.01 | *0.82* |
| *Anemia (vs. no anemia)* | 0.02 | *0.88* | -0.16 | *0.36* | -1.5x10^-3^ | *0.99* |
| *Cytoreductive therapy (vs. no cytoreductive therapy)* | 0.14 | *0.53* | 0.18 | *0.65* | 0.11 | *0.81* |
| *JAK-inhibitor therapy (vs. no JAK-inhibitor therapy)* | -0.16 | *0.33* | -0.20 | *0.44* | 0.29 | *0.59* |
| Laboratory |  |  |  |  |  |  |
| *Hemoglobin (g/dL)* | -2.6x10^-3^ | *0.94* | 0.06 | *0.23* | -0.03 | *0.67* |
| *Hematocrit (%)* | -2.2x10^-3^ | *0.84* | 0.03 | *0.19* | 4.4x10^-4^ | *0.99* |
| *Platelet count (x10^9^/L)* | 4.7x10^-4^ | *0.14* | 8.5x10^-5^ | *0.77* | -3.9x10^-4^ | *0.51* |
| *WBC count (x10^9^/L)* | -7.3x10^-3^ | *0.57* | 2.8x10^-3^ | *0.91* | **0.04** | ***<0.001*** |
| *Circulating blasts (%)* | - | *-* | - | *-* | 0.15 | *0.09* |
| *Neutrophil count (x10^9^/L)* | -0.01 | *0.51* | 4.4x10^-3^ | *0.89* | **0.06** | ***<0.001*** |
| *Ferritin (µg/dL)* | -7.1x10^-5^ | *0.93* | -2.0x10^-4^ | *0.86* | 1.4x10^-3^ | *0.17* |
| *Iron (µg/dL)* | -2.3x10^-3^ | *0.43* | -3.8x10^-3^ | *0.28* | **-0.03** | ***0.03*** |
| *TIBC (µg/dL)* | -8.2x10^-4^ | *0.66* | -1.1x10^-3^ | *0.64* | 0.01 | *0.14* |
| *Non-neutrophil WBC count (x10^9^/L)* | 0.01 | *0.86* | 0.02 | *0.82* | **0.09** | ***0.001*** |
| *Lactate dehydrogenase (IU/L)* | -1.7x10^-3^ | *0.17* | -3.7x10^-4^ | *0.60* | 5.8x10^-5^ | *0.90* |

Abbreviations: WBC: white blood cell; TIBC: total iron binding capacity.

Bold values indicate a statistically significant association with high symptom scores.

The table shows the association of demographic, clinical, and laboratory features with individual symptom scores from univariable regression in patients with polycythemia vera (PV), essential thrombocythemia (ET), and myelofibrosis (MF).

(a) In PV, depression, anemia, and low hemoglobin; in ET, depression, antidepressant consumption, and anemia; while in MF, old age, depression, anemia, low hemoglobin, high white blood cell (WBC) count, and low iron were significantly associated with fatigue.

(b) In PV, depression, high WBC and high neutrophil counts; in ET, female gender, depression, and low hemoglobin; while in MF, Hispanic/Latino ethnicity, low hemoglobin, high WBC, neutrophil and non-neutrophil WBC counts were significantly associated with early satiety.

(c) In PV, young age, female gender, and depression; in ET, depression and obesity; while in MF, depression and low platelet count were significantly associated with abdominal discomfort.

(d) In PV, female gender, depression, and low hemoglobin; in ET, non-Hispanic/Latino ethnicity, less time since diagnosis and depression; while in MF, depression, splenomegaly, and low hemoglobin were significantly associated with inactivity.

(e) In PV, depression and spleen size, while in ET and MF, having depression was significantly associated with concentration difficulties.

(f) In PV, being of non-White race; in ET, more time since diagnosis, depression, and high non-neutrophil WBC count; while in MF, having depression was significantly associated with fever.

(g) In PV, young age, female gender, and low hemoglobin; in ET, having depression; while in MF, depression, increased spleen size, and high total iron binding capacity were significantly associated with night sweats.

(h) In PV, young age and female gender; in ET, White race and depression; while in MF, female gender, non-White race, and high serum iron were significantly associated with itching.

(i) In PV, young age and female gender; in ET, having depression; while in MF, depression, obesity, high WBC count, ferritin, and non-neutrophil WBC count were significantly associated with bone pain.

(j) In PV, being of non-White race, while in MF, high WBC, neutrophil and non-neutrophil WBC count, and low iron were significantly associated with weight loss.

# **Supplementary Table 8: Predictors of Individual Symptom Scores from Multivariable Regression Analyses**

**(a) Fatigue**

| **Polycythemia vera** | | |
| --- | --- | --- |
| **Variable** | **Coefficient** | ***p-value*** |
| *Depression at the time of the visit (vs. no depression)* | **1.65** | ***0.001*** |
| *Hemoglobin (g/dL)* | -0.17 | *0.13* |
| *Anemia (vs. no anemia)* | 0.63 | *0.32* |
| **Essential thrombocythemia** | | |
| **Variable** | **Coefficient** | ***p-value*** |
| *Depression at the time of the visit (vs. no depression)* | **1.98** | ***<0.001*** |
| *Anemia (vs. no anemia)* | 1.22 | *0.02* |
| **Myelofibrosis** | | |
| **Variable** | **Coefficient** | ***p-value*** |
| *Age (years)* | 0.02 | *0.20* |
| *Depression at the time of the visit (vs. no depression)* | **2.22** | ***<0.001*** |
| *Hemoglobin (g/dL)* | **-0.45** | ***0.001*** |
| *WBC count (x10^9^/L)* | **0.03** | ***0.03*** |
| *Anemia (vs. no anemia)* | 0.01 | *0.99* |

**(b) Early satiety**

| **Polycythemia vera** | | |
| --- | --- | --- |
| **Variable** | **Coefficient** | ***p-value*** |
| *Depression at the time of the visit (vs. no depression)* | 1.19 | *0.17* |
| *WBC count (x10^9^/L)* | **0.08** | ***0.04*** |
| **Essential thrombocythemia** | | |
| **Variable** | **Coefficient** | ***p-value*** |
| *Gender: Female (vs. Male)* | 0.25 | *0.51* |
| *Depression at the time of the visit (vs. no depression)* | 0.86 | *0.35* |
| *Hemoglobin (g/dL)* | -0.11 | *0.46* |
| **Myelofibrosis** | | |
| **Variable** | **Coefficient** | ***p-value*** |
| *Ethnicity: Hispanic or Latino (vs. non-Hispanic and non-Latino)* | **2.24** | ***0.04*** |
| *Hemoglobin (g/dL)* | **-0.28** | ***0.004*** |
| *WBC count (x10^9^/L)* | **0.05** | ***0.004*** |

**(c) Abdominal discomfort**

| **Polycythemia vera** | | |
| --- | --- | --- |
| **Variable** | **Coefficient** | ***p-value*** |
| *Age (years)* | -0.01 | *0.33* |
| *Gender: Female (vs. Male)* | 0.20 | *0.39* |
| *Depression at the time of the visit (vs. no depression)* | 1.25 | *0.12* |
| **Essential thrombocythemia** | | |
| **Variable** | **Coefficient** | ***p-value*** |
| *Depression at the time of the visit (vs. no depression)* | 0.13 | *0.90* |
| *Obesity at the time of the visit (vs. no obesity)* | 3.87 | *0.06* |
| **Myelofibrosis** | | |
| **Variable** | **Coefficient** | ***p-value*** |
| *Depression at the time of the visit (vs. no depression)* | 0.63 | *0.44* |
| *Platelet count (x10^9^/L)* | -7.9x10^-4^ | *0.18* |

**(d) Inactivity**

| **Polycythemia vera** | | |
| --- | --- | --- |
| **Variable** | **Coefficient** | ***p-value*** |
| *Gender: Female (vs. Male)* | 0.44 | *0.30* |
| *Depression at the time of the visit (vs. no depression)* | **1.71** | ***0.03*** |
| *Hemoglobin (g/dL)* | -0.16 | *0.10* |
| **Essential thrombocythemia** | | |
| **Variable** | **Coefficient** | ***p-value*** |
| *Ethnicity: Hispanic or Latino (vs. non-Hispanic and non-Latino)* | **-2.35** | ***<0.001*** |
| *Time since diagnosis (months)* | **-0.01** | ***0.02*** |
| *Depression at the time of the visit (vs. no depression)* | **2.78** | ***<0.001*** |
| **Myelofibrosis** | | |
| **Variable** | **Coefficient** | ***p-value*** |
| *Depression at the time of the visit (vs. no depression)* | **2.36** | ***0.001*** |
| *Hemoglobin (g/dL)* | **-0.38** | ***<0.001*** |

**(e) Concentration difficulties**

| **Polycythemia vera** | | |
| --- | --- | --- |
| **Variable** | **Coefficient** | ***p-value*** |
| *Depression at the time of the visit (vs. no depression)* | **2.36** | ***<0.001*** |
| **Essential thrombocythemia** | | |
| **Variable** | **Coefficient** | ***p-value*** |
| *Depression at the time of the visit (vs. no depression)* | **2.67** | ***<0.001*** |
| **Myelofibrosis** | | |
| **Variable** | **Coefficient** | ***p-value*** |
| *Depression at the time of the visit (vs. no depression)* | **1.95** | ***<0.001*** |

**(f) Fever**

| **Polycythemia vera** | | |
| --- | --- | --- |
| **Variable** | **Coefficient** | ***p-value*** |
| *Race: White (vs. non-White)* | **-1.41** | ***0.008*** |
| **Essential thrombocythemia** | | |
| **Variable** | **Coefficient** | ***p-value*** |
| *Time since diagnosis (months)* | 9.4x10^-12^ | *0.99* |
| *Depression at the time of the visit (vs. no depression)* | 0.01 | *-* |
| *Non-neutrophil WBC count (x10^9^/L)* | 4.5x10^-10^ | *0.99* |
| **Myelofibrosis** | | |
| **Variable** | **Coefficient** | ***p-value*** |
| *Depression at the time of the visit (vs. no depression)* | **0.96** | ***0.04*** |

**(g) Night sweats**

| **Polycythemia vera** | | |
| --- | --- | --- |
| **Variable** | **Coefficient** | ***p-value*** |
| *Age (years)* | -0.02 | *0.09* |
| *Gender: Female (vs. Male)* | 0.41 | *0.20* |
| *Hemoglobin (g/dL)* | -0.11 | *0.12* |
| **Essential thrombocythemia** | | |
| **Variable** | **Coefficient** | ***p-value*** |
| *Depression at the time of the visit (vs. no depression)* | **1.62** | ***<0.001*** |
| **Myelofibrosis** | | |
| **Variable** | **Coefficient** | ***p-value*** |
| *Depression at the time of the visit (vs. no depression)* | **1.61** | ***0.002*** |

**(h) Itching**

| **Polycythemia vera** | | |
| --- | --- | --- |
| **Variable** | **Coefficient** | ***p-value*** |
| *Age (years)* | -0.01 | *0.38* |
| *Gender: Female (vs. Male)* | 0.22 | *0.42* |
| **Essential thrombocythemia** | | |
| **Variable** | **Coefficient** | ***p-value*** |
| *Race: White (vs. non-White)* | 5.0x10^-5^ | *0.99* |
| *Depression at the time of the visit (vs. no depression)* | 0.05 | *0.96* |
| **Myelofibrosis** | | |
| **Variable** | **Coefficient** | ***p-value*** |
| *Gender: Female (vs. Male)* | **0.58** | ***0.01*** |
| *Race: White (vs. non-White)* | -1.70 | *0.12* |

**(i) Bone pain**

| **Polycythemia vera** | | |
| --- | --- | --- |
| **Variable** | **Coefficient** | ***p-value*** |
| *Age (years)* | -2.5x10^-6^ | *0.35* |
| *Gender: Female (vs. Male)* | 6.6x10^-5^ | *0.54* |
| **Essential thrombocythemia** | | |
| **Variable** | **Coefficient** | ***p-value*** |
| *Depression at the time of the visit (vs. no depression)* | **0.57** | ***0.03*** |
| **Myelofibrosis** | | |
| **Variable** | **Coefficient** | ***p-value*** |
| *Depression at the time of the visit (vs. no depression)* | 1.3x10^-4^ | *0.99* |
| *WBC count (x10^9^/L)* | 5.8x10^-6^ | *0.99* |

**(j) Weight loss**

| **Polycythemia vera** | | |
| --- | --- | --- |
| **Variable** | **Coefficient** | ***p-value*** |
| *Race: White (vs. non-White)* | **-0.53** | ***0.01*** |
| **Myelofibrosis** | | |
| **Variable** | **Coefficient** | ***p-value*** |
| *WBC count (x10^9^/L)* | **0.04** | ***<0.001*** |

Abbreviations: WBC: white blood cell.

Bold values indicate a statistically significant association with high total symptom scores.

The table shows the association of demographic, clinical, and laboratory features with (a) fatigue, (b) early satiety, (c) abdominal discomfort, (d) inactivity, (e) concentration difficulties (f) fever, (g) night sweats, (h) itching, (i) bone pain and (j) weight loss from multivariable regression in patients with polycythemia vera (PV), essential thrombocythemia (ET), and myelofibrosis (MF). In PV, depression was associated with fatigue, inactivity, and concentration difficulties; high white blood cell (WBC) count contributed to early satiety; and non-White race contributed to fever and weight loss. In ET, depression was associated with fatigue, inactivity, concentration difficulties, night sweats, and bone pain; and non-Hispanic/Latino ethnicity and less time since diagnosis were associated with inactivity. In MF, depression was associated with fatigue, inactivity, concentration difficulties, fever, and night sweats; low hemoglobin was associated with fatigue, early satiety, and inactivity; high WBC count was associated with fatigue, early satiety, and weight loss; Hispanic/Latino ethnicity was associated with early satiety; and female gender was associated with itching.

# **Supplementary Table 9: Predictors of Symptom Scores in Patients with Depression**

**(a) Association with Total Symptom Score from Univariable Regression**

| **Total Symptom Score** | | |
| --- | --- | --- |
| ***Essential thrombocythemia*** | | |
| **Variable** | **Coefficient** | ***p-value*** |
| *Time since diagnosis (months)* | -0.10 | *0.002* |
| *TIBC (µg/dL)* | -0.47 | *<0.001* |
| ***Myelofibrosis*** | | |
| **Variable** | **Coefficient** | ***p-value*** |
| *Gender: Female (vs. Male)* | -11.86 | *0.04* |
| *Time since diagnosis (months)* | -0.16 | *0.001* |
| *WBC count (x10^9^/L)* | 0.55 | *0.01* |
| *Non-neutrophil WBC count (x10^9^/L)* | 3.72 | *0.01* |

**(b) Association with Individual Symptom Scores from Univariable Regression**

| **Fatigue** | | |
| --- | --- | --- |
| ***Polycythemia vera*** | | |
| **Variable** | **Coefficient** | ***p-value*** |
| *Antidepressants at the time of the visit (vs. no antidepressants)* | 1.87 | *0.03* |
| ***Essential thrombocythemia*** | | |
| **Variable** | **Coefficient** | ***p-value*** |
| *Time since diagnosis (months)* | -0.01 | *0.04* |
| ***Myelofibrosis*** | | |
| **Variable** | **Coefficient** | ***p-value*** |
| *Gender: Female (vs. Male)* | -2.39 | *0.02* |
| *Time since diagnosis (months)* | -0.02 | *0.02* |
| *Cytoreductive therapy (vs. no cytoreductive therapy)* | -2.6 | *0.03* |
| *JAK-i (vs. no JAK-i)* | 2.8 | *0.03* |
| *Hemoglobin (g/dL)* | -0.46 | *0.04* |
| *WBC count (x10^9^/L)* | 0.08 | *0.02* |
| *Iron (µg/dL)* | -0.07 | *0.04* |
| *Lactate dehydrogenase (IU/L)* | 0.004 | *0.01* |
| **Early Satiety** | | |
| ***Essential thrombocythemia*** | | |
| **Variable** | **Coefficient** | ***p-value*** |
| *Hematocrit (%)* | -0.30 | *0.03* |
| *TIBC (µg/dL)* | -0.06 | *0.04* |
| ***Myelofibrosis*** | | |
| **Variable** | **Coefficient** | ***p-value*** |
| *Age (years)* | -0.09 | *0.04* |
| *Ferritin (µg/dL)* | -0.01 | *0.02* |
| *Non-neutrophil WBC count (x10^9^/L)* | 0.76 | *0.002* |
| **Abdominal Discomfort** | | |
| ***Polycythemia vera*** | | |
| **Variable** | **Coefficient** | ***p-value*** |
| *Age (years)* | -0.08 | *0.04* |
| *JAK-i (vs. no JAK-i)* | 2.32 | *0.04* |
| ***Essential thrombocythemia*** | | |
| **Variable** | **Coefficient** | ***p-value*** |
| *Time since diagnosis (months)* | -0.01 | *0.02* |
| *TIBC (µg/dL)* | -0.06 | *0.02* |
| **Inactivity** | | |
| ***Essential thrombocythemia*** | | |
| **Variable** | **Coefficient** | ***p-value*** |
| *Time since diagnosis (months)* | -0.02 | *0.001* |
| ***Myelofibrosis*** | | |
| **Variable** | **Coefficient** | ***p-value*** |
| *Time since diagnosis (months)* | -0.03 | *0.01* |
| *JAK-i (vs. no JAK-i)* | 3.7 | *0.03* |
| *Neutrophil count (x10^9^/L)* | 0.14 | *0.03* |
| **Concentration Difficulties** | | |
| ***Essential thrombocythemia*** | | |
| **Variable** | **Coefficient** | ***p-value*** |
| *Neutrophil count (x10^9^/L)* | 0.45 | *0.007* |
| ***Myelofibrosis*** | | |
| **Variable** | **Coefficient** | ***p-value*** |
| *Gender: Female (vs. Male)* | -2.67 | *0.02* |
| **Fever** | | |
| ***Polycythemia vera*** | | |
| **Variable** | **Coefficient** | ***p-value*** |
| *Race: White (vs. non-White)* | -3.46 | *0.03* |
| ***Essential thrombocythemia*** | | |
| **Variable** | **Coefficient** | ***p-value*** |
| *Non-neutrophil WBC count (x10^9^/L)* | 0.89 | *0.002* |
| ***Myelofibrosis*** | | |
| **Variable** | **Coefficient** | ***p-value*** |
| *Circulating blasts (%)* | 0.57 | *0.02* |
| **Night Sweats** | | |
| ***Essential thrombocythemia*** | | |
| **Variable** | **Coefficient** | ***p-value*** |
| *Time since diagnosis (months)* | -0.02 | *0.01* |
| *TIBC (µg/dL)* | -0.08 | *0.003* |
| ***Myelofibrosis*** | | |
| **Variable** | **Coefficient** | ***p-value*** |
| *Time since diagnosis (months)* | -0.02 | *0.02* |
| *Season at the time of the visit: Summer (vs. Winter)* | -2.35 | *0.04* |
| *TIBC (µg/dL)* | 0.03 | *0.04* |
| **Itching** | | |
| ***Polycythemia vera*** | | |
| **Variable** | **Coefficient** | ***p-value*** |
| *Age (years)* | -0.10 | *0.01* |
| ***Essential thrombocythemia*** | | |
| **Variable** | **Coefficient** | ***p-value*** |
| *Ferritin (µg/dL)* | 0.01 | *0.02* |
| *TIBC (µg/dL)* | -0.08 | *0.003* |
| **Bone pain** | | |
| ***Polycythemia vera*** | | |
| **Variable** | **Coefficient** | ***p-value*** |
| *Age (years)* | -0.09 | *0.01* |
| *Gender: Female (vs. Male)* | 1.76 | *0.03* |
| *JAK-i (vs. no JAK-i)* | 2.63 | *0.01* |
| ***Myelofibrosis*** | | |
| **Variable** | **Coefficient** | ***p-value*** |
| *Obesity at the time of the visit (vs. no obesity)* | 6.35 | *0.03* |
| *Splenomegaly at the time of the visit (vs. no splenomegaly)* | 2.86 | *0.02* |
| **Weight loss** | | |
| ***Myelofibrosis*** | | |
| **Variable** | **Coefficient** | ***p-value*** |
| *Anemia (vs. no anemia)* | -2.21 | *0.04* |
| *WBC count (x10^9^/L)* | 0.10 | *0.002* |
| *Circulating blasts (%)* | 0.71 | *0.01* |
| *Neutrophil count (x10^9^/L)* | 0.17 | *0.001* |
| *Non-neutrophil WBC count (x10^9^/L)* | 0.63 | *0.01* |

**(c) Association with Total Symptom Score from Multivariable Regression**

| **Total Symptom Score** | | |
| --- | --- | --- |
| ***Essential thrombocythemia*** | | |
| **Variable** | **Coefficient** | ***p-value*** |
| *Time since diagnosis (months)* | -0.10 | *0.002* |
| ***Myelofibrosis*** | | |
| **Variable** | **Coefficient** | ***p-value*** |
| *Time since diagnosis (months)* | -0.13 | *0.004* |

**(d) Association with Individual Symptom Scores from Multivariable Regression**

| **Fatigue** | | |
| --- | --- | --- |
| ***Polycythemia vera*** | | |
| **Variable** | **Coefficient** | ***p-value*** |
| *Antidepressants at the time of the visit (vs. no antidepressants)* | 1.87 | *0.03* |
| ***Essential thrombocythemia*** | | |
| **Variable** | **Coefficient** | ***p-value*** |
| *Time since diagnosis (months)* | -0.01 | *0.04* |
| ***Myelofibrosis*** | | |
| **Variable** | **Coefficient** | ***p-value*** |
| *Gender: Female (vs. Male)* | -2.043937 | *0.01* |
| *Hemoglobin (g/dL)* | -0.61 | *0.001* |
| **Early Satiety** | | |
| ***Myelofibrosis*** | | |
| **Variable** | **Coefficient** | ***p-value*** |
| *Non-neutrophil WBC count (x10^9^/L)* | 0.71 | *0.003* |
| **Abdominal Discomfort** | | |
| ***Polycythemia vera*** | | |
| **Variable** | **Coefficient** | ***p-value*** |
| *Age (years)* | -0.08 | *0.04* |
| ***Essential thrombocythemia*** | | |
| **Variable** | **Coefficient** | ***p-value*** |
| *Time since diagnosis (months)* | -0.01 | *0.02* |
| **Inactivity** | | |
| ***Essential thrombocythemia*** | | |
| **Variable** | **Coefficient** | ***p-value*** |
| *Time since diagnosis (months)* | -0.02 | *0.001* |
| **Concentration Difficulties** | | |
| ***Myelofibrosis*** | | |
| **Variable** | **Coefficient** | ***p-value*** |
| *Gender: Female (vs. Male)* | -2.67 | *0.02* |
| **Fever** | | |
| ***Polycythemia vera*** | | |
| **Variable** | **Coefficient** | ***p-value*** |
| *Race: White (vs. non-White)* | -3.46 | *0.03* |
| ***Myelofibrosis*** | | |
| **Variable** | **Coefficient** | ***p-value*** |
| *Circulating blasts (%)* | 0.57 | *0.02* |
| **Night Sweats** | | |
| ***Essential thrombocythemia*** | | |
| **Variable** | **Coefficient** | ***p-value*** |
| *Time since diagnosis (months)* | -0.02 | *0.01* |
| ***Myelofibrosis*** | | |
| **Variable** | **Coefficient** | ***p-value*** |
| *Time since diagnosis (months)* | -0.02 | *0.01* |
| *Season at the time of the visit: Summer (vs. Winter)* | 2.50 | *0.01* |
| **Itching** | | |
| ***Polycythemia vera*** | | |
| **Variable** | **Coefficient** | ***p-value*** |
| *Age (years)* | -0.10 | *0.01* |
| **Bone pain** | | |
| ***Polycythemia vera*** | | |
| **Variable** | **Coefficient** | ***p-value*** |
| *Age (years)* | -0.08 | *0.02* |
| ***Myelofibrosis*** | | |
| **Variable** | **Coefficient** | ***p-value*** |
| *Obesity at the time of the visit (vs. no obesity)* | 6.35 | *0.03* |
| **Weight loss** | | |
| ***Myelofibrosis*** | | |
| **Variable** | **Coefficient** | ***p-value*** |
| *WBC count (x10^9^/L)* | 0.09 | *0.004* |

Abbreviations: WBC: white blood cell; TIBC: total iron binding capacity.

The table shows demographic, clinical, and laboratory variables having statistically significant association with the total (TSS) and individual symptom scores in the subset of patients with depression.

(a) Univariable regression showed less time since diagnosis in ET and male gender, less time since diagnosis and high total and non-neutrophil WBC counts in MF to have a significant association with a high TSS.

(b) Univariable regression showed young age in PV, less time since diagnosis in ET, and male gender, less time since diagnosis, high white blood cell (WBC) count, high non-neutrophil WBC counts, and high circulating blasts in MF to have a significant association with multiple symptoms.

(c) Multivariable regression showed less time since diagnosis in ET and MF to have a significant association with a high TSS.

(d) Multivariable regression analyses showed young age in PV, less time since diagnosis in ET, and male gender in MF to have a significant association with multiple symptoms.

# **Supplementary Figure 1: Residual Distribution for the Total Symptom Score**

**
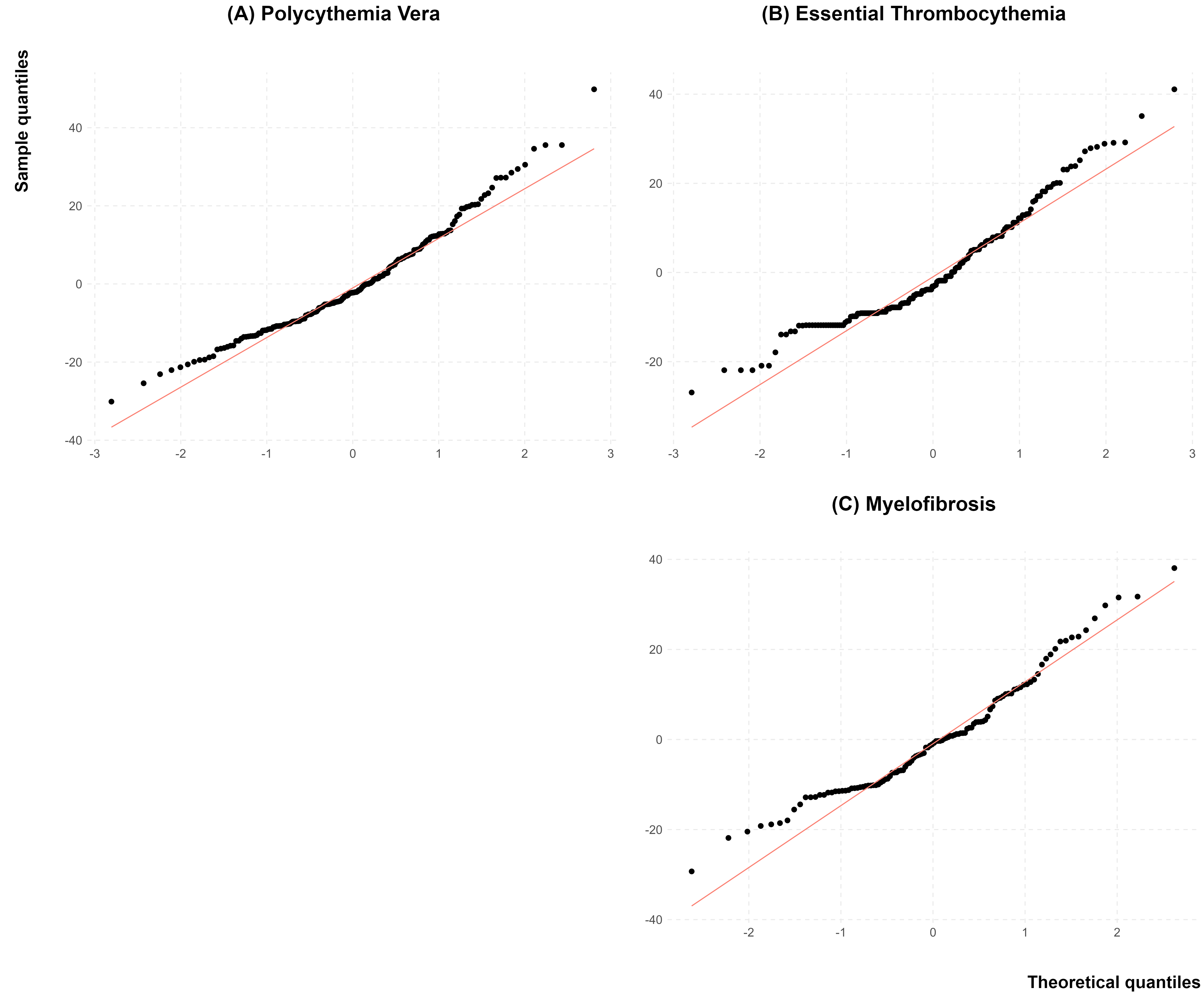
**

The figure shows QQ plots showcasing the distribution of residuals for TSS in (A) polycythemia vera, (B) essential thrombocythemia, and (c) myelofibrosis from multivariable regression analysis.

# **Supplementary Figure 2: Frequency of Individual Symptom Scores**

**
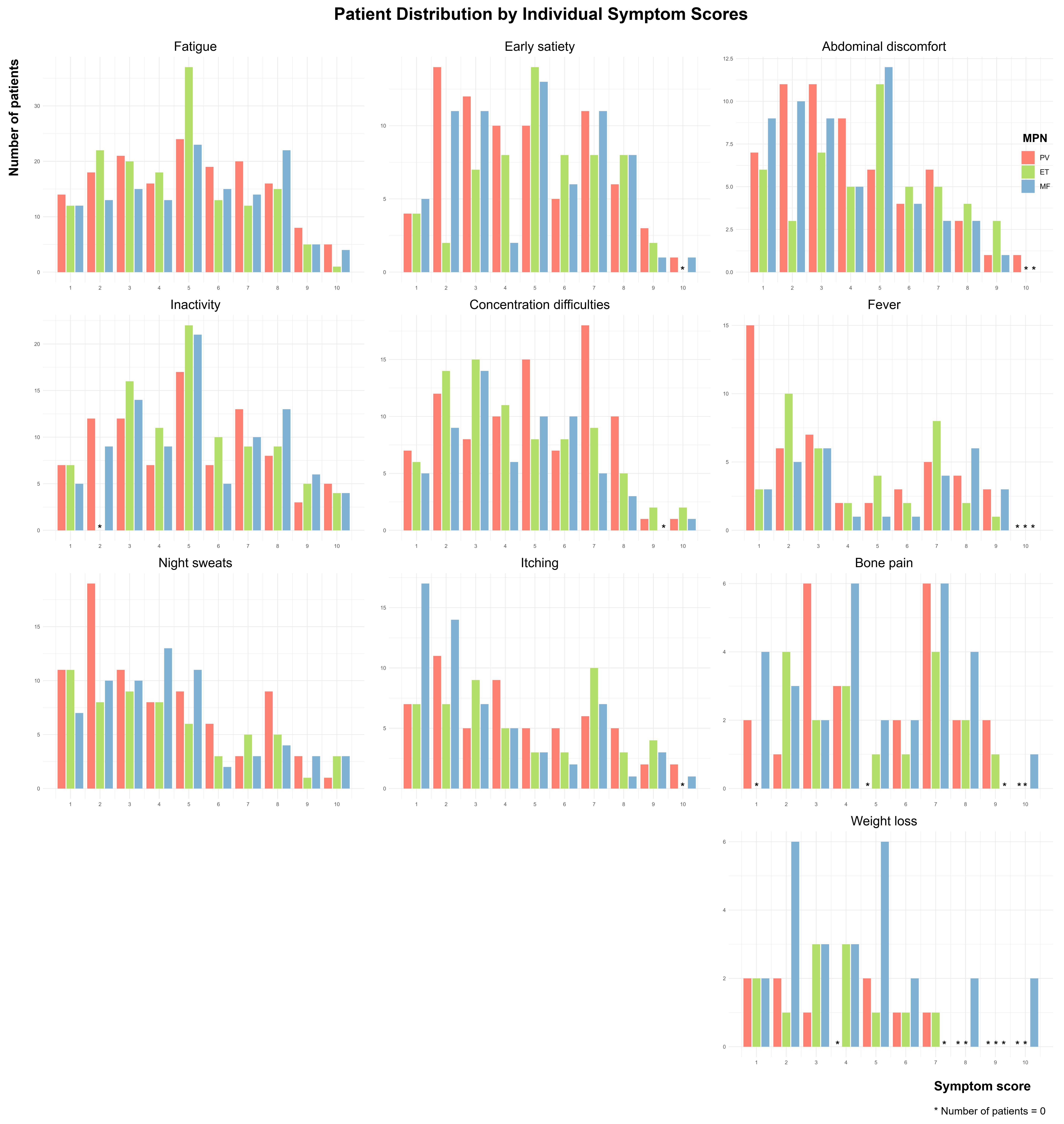
**

Abbreviations: MPN: myeloproliferative neoplasm; PV: polycythemia vera; ET: essential thrombocythemia; MF: myelofibrosis.

The figure shows the distribution of patients by individual symptom scores (0-10). Fatigue is the most common symptom for all grades of severity.
